# Incorporating a brief intervention for personalised cancer risk assessment to promote behaviour change into primary care: a multi-methods pilot study

**DOI:** 10.1186/s12889-021-10210-3

**Published:** 2021-01-23

**Authors:** Katie Mills, Ben Paxton, Fiona M. Walter, Simon J. Griffin, Stephen Sutton, Juliet A. Usher-Smith

**Affiliations:** 1 The Primary Care Unit, Department of Public Health and Primary Care, University of Cambridge School of Clinical Medicine, Box 113 Cambridge Biomedical Campus, Cambridge, CB2 0SR UK; 2 University of Cambridge School of Clinical Medicine, Addenbrooke’s Hospital, Hills Road, Cambridge, CB2 0SP UK

**Keywords:** Cancer, Risk assessment, Behaviour change, Primary care, Pilot study

## Abstract

**Background:**

Approximately 40% of cancers could be prevented if people lived healthier lifestyles. We have developed a theory-based brief intervention to share personalised cancer risk information and promote behaviour change within primary care. This study aimed to assess the feasibility and acceptability of incorporating this intervention into primary care consultations.

**Method:**

Patients eligible for an NHS Health Check or annual chronic disease review at five general practices were invited to participate in a non-randomised pilot study. In addition to the NHS Health Check or chronic disease review, those receiving the intervention were provided with their estimated risk of developing the most common preventable cancers alongside tailored behaviour change advice. Patients completed online questionnaires at baseline, immediately post-consultation and at 3-month follow-up. Consultations were audio/video recorded. Patients (*n* = 12) and healthcare professionals (HCPs) (*n* = 7) participated in post-intervention qualitative interviews that were analysed using thematic analysis.

**Results:**

62 patients took part. Thirty-four attended for an NHS Health Check plus the intervention; 7 for a standard NHS Health Check; 16 for a chronic disease review plus the intervention; and 5 for a standard chronic disease review. The mean time for delivery of the intervention was 9.6 min (SD 3) within NHS Health Checks and 9 min (SD 4) within chronic disease reviews. Fidelity of delivery of the intervention was high. Data from the questionnaires demonstrates potential improvements in health-related behaviours following the intervention. Patients receiving the intervention found the cancer risk information and lifestyle advice understandable, useful and motivating. HCPs felt that the intervention fitted well within NHS Health Checks and facilitated conversations around behaviour change. Integrating the intervention within chronic disease reviews was more challenging.

**Conclusions:**

Incorporating a risk-based intervention to promote behaviour change for cancer prevention into primary care consultations is feasible and acceptable to both patients and HCPs. A randomised trial is now needed to assess the effect on health behaviours. When designing that trial, and other prevention activities within primary care, it is necessary to consider challenges around patient recruitment, the HCP contact time needed for delivery of interventions, and how best to integrate discussions about disease risk within routine care.

**Supplementary Information:**

The online version contains supplementary material available at 10.1186/s12889-021-10210-3.

## Background

The number of new cancer cases is estimated to have reached approximately 300,000 per year in the UK by 2020 [1]. As many of these cancers may be preventable through reductions in lifestyle risk factors such as smoking, obesity and alcohol consumption [2], health promotion is increasingly becoming the focus for policy and clinical interventions. Alongside strategies to engage clinicians and commissioners, interventions to increase public awareness of cancer risk factors and promote behaviour change are required.

In the UK, primary healthcare already delivers the largest disease prevention initiative, the NHS Health Check programme, and also provides annual reviews for individuals with chronic diseases. Within the NHS Health Check programme, eligible individuals aged 40–74 are invited to attend a Health Check every 5 years. The Health Check itself consists of three components: risk assessment, risk communication and risk management [3]. Risk assessment tools are used to estimate the individual’s risk of developing cardiovascular disease (CVD) and diabetes. That assessment is then used to raise awareness of relevant risk factors and inform discussion on the lifestyle and medical approaches best suited to managing the individual’s disease risk. Chronic disease reviews for diabetes, asthma, hypertension and chronic obstructive pulmonary disease include assessment of the current condition, review of blood tests and current medication and discussion about associated lifestyle factors.

As many of the risk factors for the most common preventable cancers (such as lung, colorectal and breast cancer) are the same as those for CVD and chronic diseases, the NHS Health Check programme and chronic disease reviews provide an ideal opportunity to deliver interventions encouraging behaviour change for cancer prevention to large numbers of the population. The inclusion of cancer may also increase the impact of the NHS Health Check on CVD and diabetes: while studies of the impact of provision of CVD and diabetes risk suggest that risk information influences decisions around medication but not health-related behaviours [4], there is greater uncertainty surrounding the impact of cancer risk information [5]. Furthermore, recent studies have reported that both patients [6] and primary care clinicians [7] would welcome the inclusion of cancer into both NHS Health Checks and chronic disease reviews, with primary care clinicians reporting that discussions about cancer are part of their working roles and primary care is an appropriate setting for these.

Using behaviour change theory, reviews of existing literature and expert opinion, we have developed a brief intervention (the I-CaPP intervention) to facilitate discussions about cancer risk and prevention within primary care [8]. In focus group discussions and online usability testing with 65 healthcare professionals (HCPs) currently involved in prevention activities in primary care within one Clinical Commissioning Group in the East of England, the intervention prototype was described as potentially acceptable and one that would fit within current practice and may encourage patients to accept recommendations and motivate them to change their behaviour [8]. However, there is a need to assess the feasibility of its delivery in practice. In particular, concerns were expressed about the additional consultation time and resources required.

The aim of this pilot study was to assess the feasibility and acceptability, both to HCPs and patients, of implementing this brief intervention incorporating cancer risk information to promote behaviour change within NHS Health Checks and chronic disease reviews in primary care.

## Methods

### Study design

The study was a mixed-methods non-randomised pilot study [9]. To enable us to compare the recruitment of participants and feasibility of the delivery of the intervention in both NHS Health Checks and chronic disease reviews, we recruited patients into several study groups. Two groups received the standard NHS Health Check or chronic disease review and two groups received either an NHS Health Check or chronic disease review plus the I-CaPP intervention. In all groups, clinical measurements and lifestyle information were collected as in usual care.

### Study patients and recruitment

In order to enable us to observe any differences between practices and have a sufficient pool of participants to recruit for the qualitative interview component of the study, we planned to recruit five general practices and 80 participants, 60 of whom would receive the intervention. The NIHR Clinical Research Network: Eastern supported recruitment of the five general practices. Three additional practices were also recruited but they withdrew before the start of patient recruitment due to other commitments. The profile of each practice was obtained from the Public Health England General Practice profile online database [10], and the decile of deprivation extracted based on the Indices of Deprivation document published by the UK government in September 2019 [11], with the lowest decile indicating the most deprived population. Additional File 1 shows the characteristics of each of the five practices. All practices were about the same size as or larger than the national average practice (8726 patients) [12]. The deprivation index for each practice ranged from six to 10, with four of the five practices in the two least deprived deciles. All served a predominantly white population.

Potentially eligible patients were identified from electronic searches already in use by the practices to identify patients eligible for NHS Health Checks or chronic disease reviews. These searches were conducted by each practice, with a GP screening potential patients to ensure eligibility prior to the invitation. Patients are eligible for an NHS Health Check if they are between the ages of 40–74, do not have a diagnosis of CVD, diabetes, kidney disease or hypertension, and have not had an NHS Health Check within the past 5 years. Patients are eligible for a chronic disease review if they have been diagnosed with the relevant disease and have not had a review in the previous 12 months. Eligible patients for this study were those over 40 years of age who were due a NHS Health Check or annual chronic disease review, did not have a current diagnosis or medical history of cancer or dementia, were not known to suffer from psycho-social issues or severe illness, in the GP’s clinical opinion had a life expectancy of more than 1 year, and were able to provide written informed consent. Patients not meeting all these eligibility criteria were excluded from the study.

**Fig. 1:**
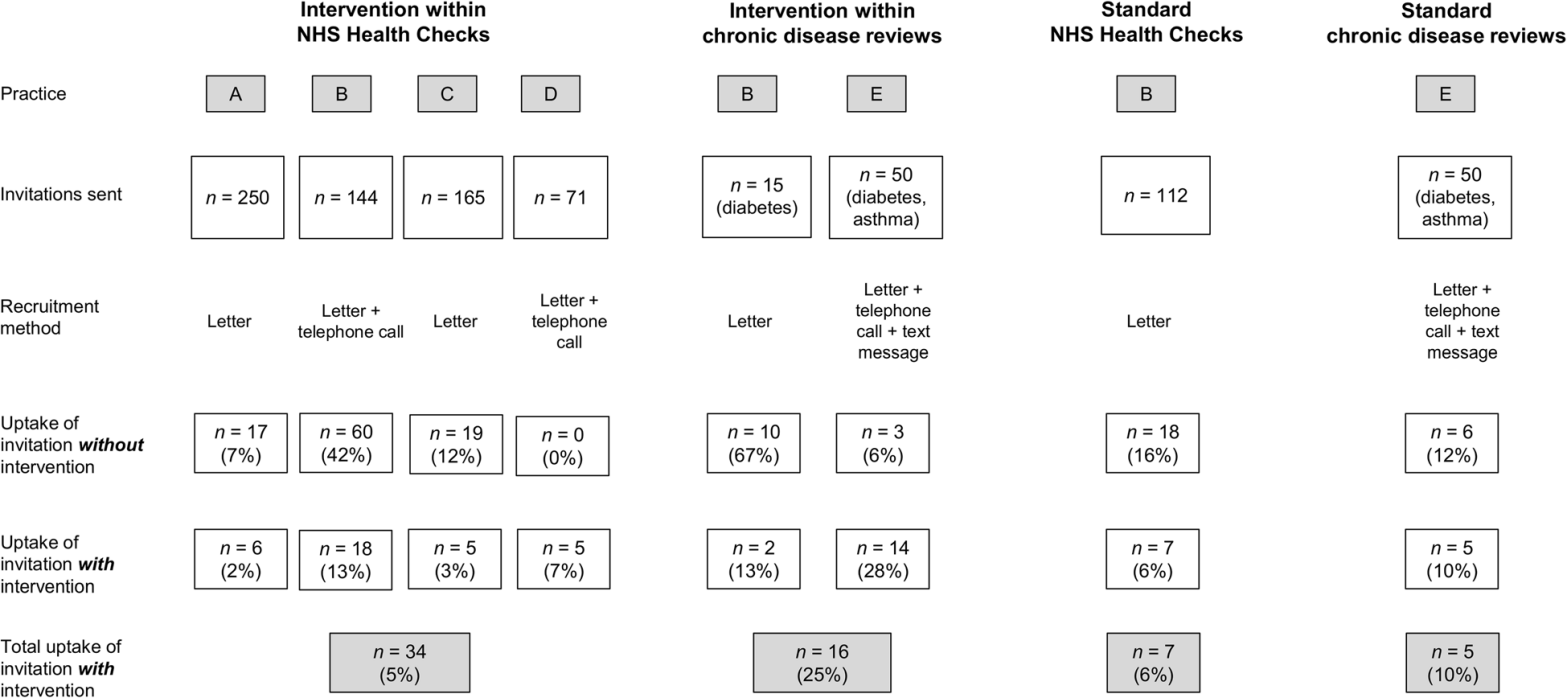
Recruitment flow chart

Eligible patients were invited into the study by invitation in the usual method by their general practice (Fig. 1). These varied by practice and included sending personalised letters via post with or without follow-up with a telephone call by practice administrative staff or text message reminder (Additional File 1 and Fig. 1). Study clinics were held on different days of the week and included the morning and afternoon sessions to facilitate attendance.

### I-CaPP intervention

The I-CaPP intervention is modelled on the three components of the NHS Health Check. The risk assessment and risk communication components comprise an online risk assessment tool that is used within the consultation to provide the patient with an estimated 10-year risk of developing one of the most common preventable cancers based on their current lifestyle [9]. The common preventable cancers are specific to each sex. For women they are breast, lung, colorectal, endometrial and kidney cancer, and for men they are lung, colorectal, bladder, kidney and oesophageal cancer. The risk management component includes guidelines for the HCPs to deliver lifestyle advice for risk modification, including the opportunity for the patient and HCP together to set targets and see a revised risk estimate. At the end of the consultation, the patient is then given a behaviour change leaflet, a copy of the risk assessment, and website log-in details to enable them to re-visit the website post-consultation.

### Practice set-up and training

Practice set-up included an initial meeting between the research team and practice staff, including both clinical and administrative teams, and a 30-min face-to-face training session for all HCPs involved in the delivery of the intervention. Nine HCPs (4 practice nurses and 5 healthcare assistants) were trained to deliver the intervention. At four of the five practices two HCPs delivered the intervention, at one practice one HCP delivered the intervention. During the recruitment period two HCPs left their roles at the practice and so recruitment continued but with less consultation and resource availability at these practices. Training focused initially on risk factors for cancer, highlighting the overlap between these and CVD. It covered the rationale behind the inclusion of the intervention, to share cancer risk and promote behaviour change, and how this could be included within existing consultations. Guidance was then given on the use of the intervention, including practising with test patients to allow for familiarisation with the website and behaviour change leaflet. Written step-by-step guidance was also provided as a reference (Additional File 2).

### Data collection

We collected data from four sources:

#### 1. Audio/video recordings of patient consultations

With consent from both patients and HCPs, the study consultations were video or audio recorded to capture the delivery of the intervention.

#### 2. Patient and HCP interviews

Patients who had received, and HCPs who had delivered, the intervention were invited to participate in semi-structured audio-recorded interviews. During the patients’ interviews, the discussion explored the impact of the intervention, particularly on perceived risk, response-efficacy and intentions to make behaviour change, alongside thoughts on the format, content and delivery of the intervention within the consultation (Additional File 3). The interviews with HCPs focused on the format, content and delivery of the intervention, the training received and the potential barriers and facilitators to implementation (Additional File 4).

#### 3. Patient questionnaires

Patients completed three online questionnaires; at baseline, immediately post-consultation and at 3-month follow up. The questionnaires were specific to each patient group and included information on demographics, lifestyle risk factors and family history of CVD and cancer as well as validated measures of numeracy [13], time orientation [14], self-rated general health, CVD risk perception, cancer risk perception [15], cancer risk awareness [16], cancer-related worry [17, 18], anxiety [19], maladaptive coping [20], self-efficacy [21] and response efficacy [22] (Additional Files 5, 6, 7). Index of multiple deprivation (IMD) was derived from each participant’s home postcode and grouped into quintiles using 2019 English indices of deprivation data [11].

#### 4. Process data from the intervention website

We collected quantitative data from the website on the time spent completing the questionnaires, the interaction with the website during the intervention, and whether patients returned to the web-based information after the consultation.

### Consent

#### Patient consent

Patients provided online consent to complete the baseline questionnaire prior to the consultation. Written consent was then sought from each patient immediately before the consultation. This included consent for the audio or video recording of the consultation and the post-consultation and 3-month follow-up questionnaires. For the patients who agreed to have their consultation recorded, a confirmation of this consent was also taken on completion of the consultation. Patients who took part in qualitative interviews completed written consent prior to commencement of the interview.

#### HCP consent

Written informed consent was obtained from each HCP at the start of the study. At the end of each recruitment session, the HCPs provided additional written consent for each of the recorded consultations. The HCPs also provided written consent prior to completion of qualitative interviews at the end of the recruitment.

### Analysis

#### Audio/video recordings of patient consultations

The audio and video recordings were used to assess the fidelity of intervention delivery and the time taken to deliver the intervention. A fidelity checklist was devised based on the main elements of the intervention covered in the training session for HCPs. Using the video or audio recordings, two researchers (KM and BP) piloted the checklist with four consultations. One researcher (BP) then assessed the remaining consultations. Both mandatory and optional elements of the intervention delivery were assessed and subsequently summarised descriptively.

#### Patient and HCP interviews

Patient and HCP interviews were analysed using thematic analysis [23]. Using an iterative analytic approach from the beginning of data collection, each interview transcript was first repeatedly read by one researcher (KM, a research associate with qualitative expertise) in order to identify patterns within the data and develop a coding framework. The qualitative dataset was then fine-coded within NVivo software (QSR International, version 12) by the same researcher and the codes sorted and combined to generate themes. These initial themes were then reviewed and refined alongside two further researchers (JUS and FMW, both academic GPs with qualitative expertise) who had each read a selection of the interview transcripts.

#### Patient questionnaires

Descriptive statistics were used to summarise the baseline characteristics of the patients from the self-completion questionnaires and the follow-up rates. The potential effects of the NHS Health Check/chronic disease review alone or in combination with the I-CaPP intervention were then summarised by reporting the difference in mean values (follow-up minus baseline) with 95% confidence intervals for each continuous outcome and the proportion in each category at each time point for categorical outcomes. The views of the participants who received the intervention on the lifestyle advice and risk information provided were also summarised. As this was a feasibility study, no formal statistical tests were performed.

#### Process data from the intervention website

The data from the website on time participants spent completing the questionnaires, the pages viewed during the consultation and whether participants returned to the site after the consultation were summarised by reporting the mean with 95% confidence intervals.

## Results

### Feasibility of recruitment

In total, 62 patients were recruited between June 2018 and March 2019. The response rates for each patient group are shown in Fig. 1. Thirty-four patients (5% of those invited) attended for an NHS Health Check plus the I-CaPP intervention; 7 (6% of those invited) attended for a standard NHS Health Check; 16 (25% of those invited) attended a chronic disease review plus the I-CaPP intervention; 5 (10% of those invited) a standard chronic disease review. The characteristics of the patients in each group are detailed in Table 1. The mean age of the patients was comparable between the patient groups (56.2 years for two groups receiving the I-CaPP intervention and 55.0 years for the NHS health check/chronic disease review standard consultation groups). Fifty-two percent and 72% respectively were female, over 90% were White British, and over 88% had completed at least secondary education.

**Table 1:** Baseline characteristics of patients

	n	NHS Health check or chronic disease review plus I-CaPP intervention (***n*** = 50)	n	Standard NHS Health Check or chronic disease review (***n*** = 12)	n	Interview Group (***n*** = 12)
**Age**	50		12		12	
Mean (sd)		56.2 (11.8)		55.0 (8.3)		62.5
Range		40–84		40–75		40–83
**Sex (n, % female)**	50	26 (52)	12	9 (75)	12	6 (50)
**Ethnicity (n, % white)**	50	44 (88)	12	11 (92)	12	11 (92)
**Family history of cancer (n, %)**	50	22 (44)	–	–		7 (58)
**Education (n, %)**	50		12		12	
No formal education or Primary Education		1 (2)		2 (17)		0 (0)
Secondary Education		27 (54)		6 (50)		8 (67)
University Education		22 (44)		4 (33)		4 (33)
**Deprivation (n, %)**	50		11		12	
Least deprived 1		11 (22)		1 (9)		2 (17)
2		18 (36)		7 (64)		6 (50)
3		11 (22)		1 (9)		2 (17)
4		10 (20)		2 (18)		2 (17)
5		0 (0)		0 (0)		0 (0)
**Perceived General Health (n, %)**	50		12		12	
Very Good / Quite Good		39 (58)		10 (92)		10 (83)
Neither Good nor Poor		8 (16)		0 (0)		2 (17)
Quite Poor / Poor		3 (6)		1 (8)		0 (0)
**Numeracy (n, %)**	44		11		12	
High numeracy (≥2)		31 (70)		5 (45)		7 (58)
Low numeracy (< 2)		13 (30)		6 (55)		5 (42)
Estimated risk (mean, sd)*						
RRI	50	1.7 (0.9)	12	1.7 (0.8)	12	1.7 (1.2)
RR	50	1.0 (0.4)	12	1.1 (0.33)	12	1.01 (0.5)
Absolute risk	50	3.6 (2.3)	12	4.4 (2.2)	12	3.86 (2.33)
**Smoking status (n, %)**	50		12		12	
Never smoker		28 (56)		4 (33)		7 (58)
Ex-smoker		19 (38)		7 (58)		5 (42)
Current smoker		3 (6)		1 (8)		0 (0)
**Lifestyle (mean, sd)**						
BMI (kg/m2)	50	27.5 (5.6)	12	29.7 (8.0)		26.6 (3.2)
Alcohol (units/week)	50	9.8 (13.6)	12	6.2 (8.7)		12.1 (17.5)
Physical Activity (hours/week)	50	5.7 (7.7)	12	5.1 (8.0)		8.4 (8.3)
Fruit (portions/day)	50	2.1 (1.4)	12	2 (0.9)		1.5 (1.2)
Vegetables (portions/day)	50	2.6 (1.3)	12	2.1 (0.7)		2.8 (1.3)
Red meat (portions/week)	50	2.1 (1.3)	12	1.3 (1.1)		2.7 (1.6)
Processed meat (portions/week)	50	1.6 (1.6)	12	1 (0.7)		1.6 (1.3)
**Cancer risk perception (mean, sd)**						
Perceived absolute risk	45	29.3 (22.8)	–	–	10	41 (17.9)
Conviction of perceived absolute risk	48	3.58 (1.7)	–	–	11	3.27 (1.6)
Perceived comparative risk	50	3.48 (1.2)	–	–	12	3.41 (0.9)
Conviction of perceived comparative risk	47	3.86 (1.6)	–	–	12	3.08 (1.7)
**Absolute risk accuracy (n, %)**	45		–		10	
Accurate (±5%)		8 (18)		–		0 (0)
Underestimate		2 (4)		–		0 (0)
Overestimate		35 (78)		–		10(100)
**Comparative risk accuracy (n, %)**	50		–		12	
Accurate		18 (36)		–		4 (33)
Underestimate		24 (48)		–		7 (58)
Overestimate		8 (16)		–		1 (8)
**Cancer Worry (mean, sd)**	46	4.8 (2.0)	–	–	10	5.4 (2.1)
**Anxiety (mean, sd)**	45	13.1 (2.2)	–	–	11	13.81 (2.0)
**Cancer risk factor awareness**	43	40.9 (5.5)	–	–	10	42.8 (4.9)

RRI - Risk relative to an individual of the same age and sex with a recommended lifestyle and RR- Risk relative to an individual of the same age and sex . *All risk estimates represent the 10 year risk fo developing one of the five most common, gender specific, preventable cancers

One patient withdrew from the study during the study consultation, and another withdrew prior to completion of the 3-month follow-up. Three did not consent to audio or video recording. Seventy-percent (*n* = 44/62) of patients completed the immediate follow-up questionnaire and 60% (*n* = 37/62) the questionnaire at 3-months. There were no marked differences between the characteristics of those who completed and did not complete the questionnaires at each time point of the study.

### Feasibility of delivering the intervention

The mean duration of the consultations in each group are given in Table 2. There was variation between practices in the time taken for both the standard consultations and the time to deliver the intervention. The mean time taken for delivery of the I-CaPP intervention within the NHS Health Check was 9.6 min (SD 3, range 3.1–15.1) and within the chronic disease reviews was 9 min (SD 4, range 4.5–15.5). As a consequence of this time taken for delivery of the intervention, the mean duration of the NHS Health Check plus I-CaPP intervention was 23.6 min (SD 7, range 14.1–39.6), and 32.4 min (SD 14, range 11.1–62.8) in the chronic disease review plus I-CaPP intervention consultations.

**Table 2:** Length of consultations and use of website within consultations

				Practice A		Practice B		Practice C		Practice D		Practice E
	n	Mean (SD) range / n (%)	n	Mean (SD) range / n (%)	n	Mean (SD) range / n (%)	n	Mean (SD) range / n (%)	n	Mean (SD range / n (%)	n	Mean (SD) range / n (%)
**Length of I-CaPP intervention delivery within NHS Health Check (min)**	32	9.6 (3.2) 3.1–15.1	6	7.5 (2.5) 4.9–12.3	17	10.4 (3.0) 6.1–15.1	4	11.5 (1.9) 8.8–13.0	5	7.6 (3.8) 3.1–13.5	–	–
**Length of I-CaPP intervention delivery within chronic disease review (min)**	14	9.0 (3.6) 4.5–15.5	–	–	–	–	–	–	2	6.8 (0.7) 6.3–7.3	12	9.4 (3.7) 4.5–15.5
**Lifestyle pages viewed in consultation**	49		6		18		5		8		13	
0		17 (35)		1 (17)		6 (33)		0 (0)		2 (0)		8 (62)
1		10 (20)		1 (17)		2 (11)		2 (40)		3 (43)		2 (15)
2–3		19 (39)		4 (67)		10 (56)		1 (20)		1 (14)		3 (23)
> 3		3 (6)		0 (0)		0 (0)		2 (40)		1 (14)		0 (0)
**Targets set during consultation**	49		6		18		5		7		13	
0		5 (10)		3 (50)		0 (0)		0 (0)		1 (14)		1 (8)
1		19 (38)		1 (17)		11 (61)		3 (60)		3 (43)		1 (8)
2–3		17 (35)		1 (17)		6 (33)		0 (0)		1 (14)		9 (69)
> 3		8 (16)		1 (17)		1 (6)		2 (40)		2 (29)		2 (15)
**Goals set during consultation**	49		6		18		5		7		13	
0		44 (90)		4 (67)		17 (94)		5 (100)		7 (100)		2 (15)
1		5 (10)		2 (33)		1 (6)		0 (0)		0 (0)		11 (85)

**Fig. 2:**
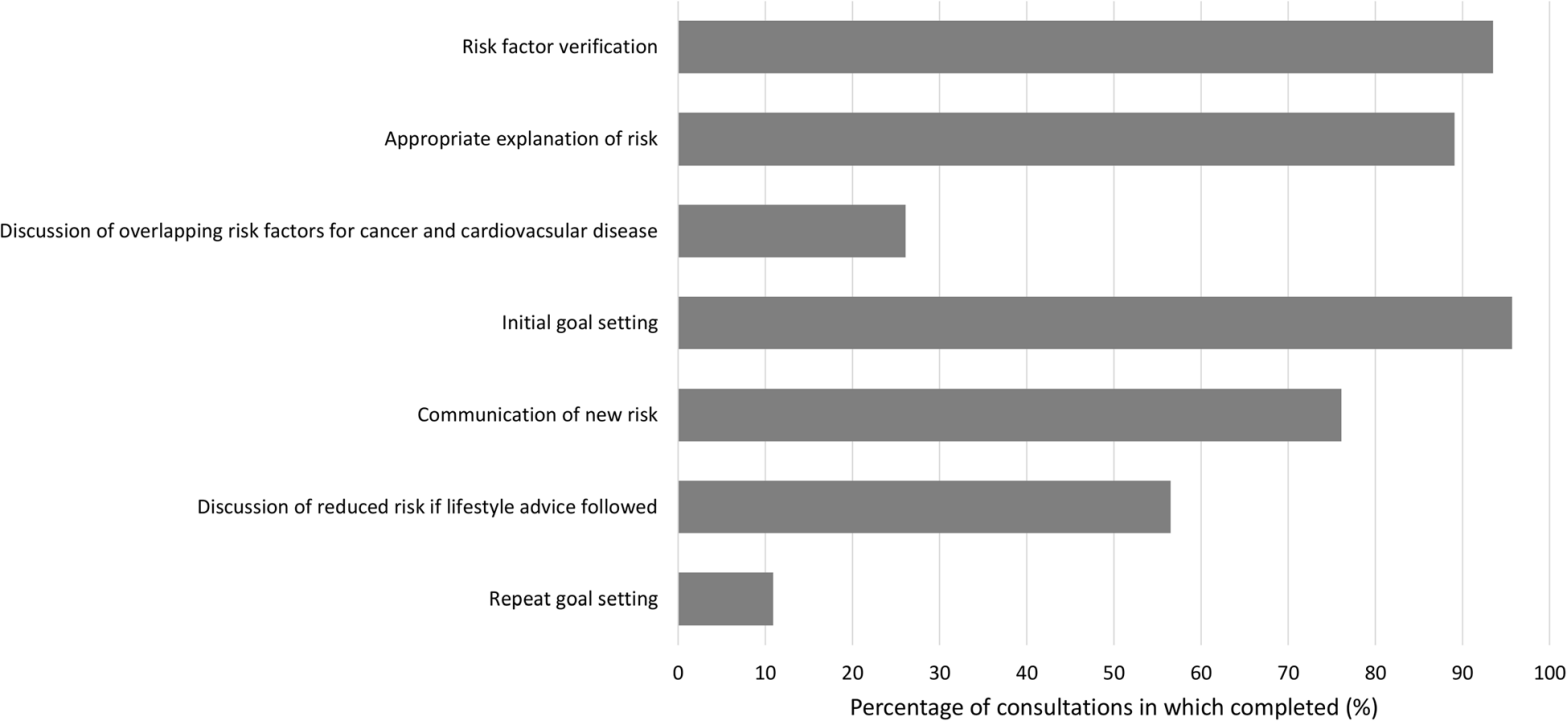
Fidelity of intervention delivery

Based on the 46 consultations that were audio or video recorded, overall the fidelity of delivery of the intervention was high (Fig. 2). HCPs verified patient responses to the risk factor questions prior to the risk calculation in 43/46 (94%) of consultations and, although amendments were made in 84% of consultations, these were small and less than one unit for each risk factor. The HCPs used a variety of descriptions of the risk presentation, with 89% using phrases in keeping with the intervention delivery training such as *“This is based on people who are like you, this is not necessarily yourself – it’s people with your height, weight, ethnic category - those sort of things”*. Initial goal setting was completed with 96% of patients, with the new risk being communicated to 76%. For those patients with risk scores in line with those following the lifestyle guidance, HCPs tended to encourage them to continue with their current lifestyle and so there was less requirement for completion of target setting. In some cases, the HCPs continued to complete this section suggesting small improvements to lifestyle to ensure that the risk level was optimised. Despite inclusion in the training, only 26% of consultations included a discussion of the overlap between the risk factors for cancer and cardiovascular disease. Optional features of the intervention that HCPs used included the display of percentage risk (59%), lifestyle advice accessible via the hyperlinks provided on the website (48%) and the option to write goals into the website (11%).

### Acceptability of the intervention to HCPs

Seven HCPs (3 practice nurses and 4 healthcare assistants, with at least one from each included practice) participated in interviews. Six themes (in italics below) were identified (Table 3). In general, HCPs were positive about the overall *delivery of the intervention* and the specific components. Many were encouraged by the potential for the intervention to promote behaviour change with specific reference to the ease of communication of the risk presentation. Discussion also focused on the ease of *patient engagement* in a shared activity, and using the computer to view the potential effects of lifestyle changes on risk of cancer. All felt this to be the most effective element of the intervention. Alongside the patient’s current risk, demonstrating a change in risk visually on the screen enabled the HCPs to introduce specific *behaviour changes* and show their impact in real time. HCPs found sharing lifestyle information via the website and providing a written leaflet to be useful additional resources for patients to consider after the consultation.

**Table 3:** Illustrative quotes of healthcare professional views

Qualitative theme	Illustrative quotes
**Delivery of the intervention**	*“I think because initially you tell them, it’s not them, it’s people like them, they take that nicer than if you said this is your risk of cancer, so yeah, a lot better than I ever thought that they would ever accept that risk” HCP 2*
**Patient engagement**	*“It was good to share the information and to be able to actually see it on the screen. The patients appreciated being able to see everything and being able to change different bits to show them how their risk was going to change” HCP 3* *It’s nice to both sit there together and share the screen, sort of discuss what we could see” HCP 1*
**Behaviour changes**	*“It seemed to ping in their heads and they thought, “actually no, I can do this and there’s a good reason for doing it”, which with years of talking to them about diabetes I haven’t noticed that reaction so quickly.” HCP 6* *“We had a patient that within one of those health checks we did, that actually gave up smoking. I gave them an inhalator in the consultation, and I’ve seen them since and they’ve actually quit smoking” HCP 2* *“I think it is a good backup. I think something written is always good as what you say in your consultation they don’t always remember. So even if it is just a case of the front page and starring “quitting smoking” and “reducing alcohol” then it is something for them to go back to. Or if it was someone else asked them they would get it out and produce it” HCP 7* *“I think when you tell people about cardiovascular health and then you explain it’s about heart attacks and strokes, I don’t think they necessarily take that on board as much because they assume it’s the people who smoke and drink. So they just brush over the cardiovascular and then when you go into cancer, they go right okay, and they sit up and pay attention” HCP 2*
**Implementation into normal practice**	*“I found it worked really well, and I also felt that actually it really enhanced what you were doing with the diabetes” HCP 6* *“Because the patients (diabetics), they really want to know about their sugar and their cholesterol, or the management of the blood sugars and treatment. So I think you’d have to introduce that (intervention) at the beginning of the consultation.” HCP 4* *“…pulling it out of thin air would be scary but I think if you’re putting it in context with something else, you can get away with it. So if it’s within a different type of check that they’re having, I don’t know COPD or for something else” HCP 2* *“So, when you talk about alcohol, it would sort of incorporate both the Health Check and the cancer part of it. It would be nice if it flowed more in that way, because you’re saying one minute that this is what you can drink is okay for the NHS Health Check, but actually this (intervention) is saying ideally you wouldn’t be drinking (alcohol)” HCP 1* *“I think it does separate it a little bit. I suppose if it was integrated into the actual template and health check, if they could be done in some way, then perhaps that would be easier” HCP 4*
**Risk presentation**	*“I think the colours work more than the numbers to be honest. Yes, if you see red you think “right ok I’m in trouble and need to do something here” HCP 7* *“I think the patients just go “Oh that’s alright then, it’s only one of 2 %”, even it’s red on the picture and you’re trying to say that you need to work on it. When you get the percentages up, they’re like, “Ah, I’m not worried”. HCP 1*

The addition of cancer risk information was described by some HCPs as having an impact on patients’ responses to risk information and willingness to consider lifestyle changes. Many felt the format of the *risk presentation* to be of huge value in communicating information about risk of cancer. Some highlighted how the colour coding displayed on the graph enhanced patient understanding. Including the percentage risk was felt to be less helpful as most HCPs described how the scores were interpreted by patients to indicate low risk and hence not something to be concerned about.

The intervention was reported by most to be suitable for inclusion in NHS Health Checks but there were mixed views on its integration within chronic disease reviews. To ensure effective *implementation into normal practice* within NHS Health Checks and chronic disease reviews, the HCPs considered factors that would be of benefit. These included integration in the practice computer software and a more holistic conversation around risk of diabetes, cancer and cardiovascular disease and associated behaviour change. Additional suggestions to refine the intervention training included greater provision of information on specific types of cancer.

### Acceptability of the intervention to patients

Twelve patients (Table 1) participated in interviews. Six themes (in italics below) were identified (Table 4). Prior to the delivery of the intervention, some patients reported *knowledge of the risk factors for cancer*. They referred to specific risk factors that did not apply to them such as cigarette smoking, which they felt ensured that their risk was low for both CVD and cancer. Patients reflected on their *expectations of their risk*. Most felt that the risk level presented was as expected but seeing it made them focus on the impact of their lifestyle choices on their risk of cancer. The *risk presentation* was generally quite well understood and they felt that the format facilitated interpretation. All described how the colour coded graph on its own or alongside the display of the risk percentage was helpful. This was in contrast to the communication of CVD risk which was noted to only be delivered verbally as a percentage. The visual display of the *risk modification* was reported by some patients to act as a motivator for behaviour change. Among those patients, those with a relatively low risk reported feeling motivated to maintain their current lifestyle and those who needed to make *behaviour change* felt better informed and had been provided with suggestions on how to make small changes to reduce their future risk. The patients also expressed how the provision of cancer risk information within the NHS Health Check was acceptable to them and fitted well with CVD risk communication.

**Table 4:** Illustrative quotes of patient views

Qualitative theme	Illustrative quotes
**Knowledge of the risk factors for cancer**	*“I mean well from my knowledge of cancer and probably the general public knowledge of cancer, I know there are certain things that you can do to help lower your risk” Patient 6* *“I knew that I didn’t smoke and I don’t drink a lot (alcohol) so I knew the ones most people get because of their lifestyle” Patient 1*
**Expectations of their risk**	*“I suppose that’s roughly where I probably thought I would be. But when it’s actually stuck in front of you like that, it focuses the mind a bit more.” Patient 8* *“While I was aware of the sort of things I should be doing in my lifestyle I didn’t really appreciate what the cancer impact of those choices could have” Patient 9*
**Risk presentation**	*“I found it very easy to understand. It shows you- projected, what you could do, and it tells you that in a very straightforward way, I thought” Patient 11* *“So, by showing you in a chart, you can understand it better, even if you haven’t got a technical mind” Patient 5*
**Risk modification**	*“It’s made me more aware of trying to do these things, especially the losing weight, side of it, which is the most difficult part, to be quite honest” Patient 2* *“That’s the thing that really hit me more than anything when that came up. And by just discussing with me a little bit, if I change my lifestyle a bit this way, that way, she showed me how it would head more towards green” Patient 8*
**Behaviour change**	*“I think that merely going through this exercise has helped to push my motivators in the right direction” Patient 7* *“I’d been smoking, prior to then, when I came out of there, I made a change straight away” Patient 5* *“Now I’m aware, it’s on my mind all the time. Like when we were away, I tried to eat chicken as opposed to steaks and that sort of thing” Patient 10*
**Provision of cancer risk information**	*“…there’s a good advice about your healthy lifestyle or your choices about getting a healthy lifestyle which will benefit both your cardiovascular and the chances of getting cancer. So I think it compliments each other really well. I was happy to have the information in one go because you can look at the way you’re living and your life and then you can make decisions” Patient 11*

Patients’ views on the risk information and lifestyle advice are summarised in Table 5. Overall, patients agreed or strongly agreed that the lifestyle advice presented in the intervention was understandable (85%), trustworthy, (76%), useful (82%), motivating (80%), important (72%) and well-presented (79%). The risk information was also felt to be understandable (94%) and trustworthy (89%). Four patients re-visited the website. All who re-visited entered additional targets into the website to view the impact of further modifications on their future risk of cancer. Two patients also viewed the lifestyle information webpages.

**Table 5:** Views of patients who received the intervention on the lifestyle advice and risk information

	***N***	Strongly disagree ***n*** (%)	Disagree ***n*** (%)	Neither ***n*** (%)	Agree ***n*** (%)	Strongly agree ***n*** (%)
**Lifestyle Advice**						
1 Understandable	34	0 (0)	1 (3)	4 (12)	17 (50)	12 (35)
2 Trustworthy	34	0 (0)	0 (0)	8 (24)	15 (44)	11(32)
3 Useful	34	0 (0)	0 (0)	6 (18)	17 (50)	11 (32)
4 Motivating	30	0 (0)	1 (3)	5 (17)	14 (47)	10 (33)
5 Important	29	0 (0)	1 (3)	7 (24)	9 (31)	12 (41)
6 Well presented	29	0 (0)	2 (7)	4 (14)	12 (41)	11 (38)
**Risk Information**						
1 Understandable	35	1 (3)	0 (0)	1 (3)	21 (60)	12 (34)
2 Trustworthy	35	1 (3)	0 (0)	3 (9)	21 (60)	10 (29)
3 Useful	35	1 (3)	1 (3)	1 (3)	19 (54)	13 (37)
4 Motivating	32	0 (0)	1 (3)	4 (13)	17 (53)	10 (31)
5 Important	32	0 (0)	1 (3)	5 (16)	16 (50)	10 (31)
6 Well presented	32	0 (0)	2 (6)	4 (13)	14 (44)	12 (38)

### Potential effects of the intervention

Immediately after the intervention, participants reported high levels of intention to change behaviour across all the risk factors discussed (Table 6). There were also several potential effects on lifestyle factors and modelled risk of cancer at 3 month follow-up (Table 7). In particular, amongst those who received the intervention, reported fruit consumption increased (mean change 0.67 portions per day, 95% CI 0.1 to 1.23), and there were decreases in red meat and processed meat consumption (mean change 0.47 portions per week, 95% CI − 0.87 to − 0.09; and − 0.35 portions per week 95% CI − 0.68 to - 0.02 respectively). There was also a reduction in estimated relative risk in comparison to the average person (mean change − 0.34, 95% CI − 0.56 to − 1.28). These changes were not observed in those who received the standard consultations without the I-CaPP intervention. These changes were accompanied by an increased awareness of cancer risk factors immediately following the consultation (mean change 2.9 95% CI 0.10 to 5.0) and 3 months post consultation (mean change 1.58, 95% CI 0.04 to 3.13). There was no evidence that the intervention increased levels of anxiety or worry or that it improved accuracy of risk perception (Additional File 8).

**Table 6:** Intention to change behaviour immediately post intervention delivery

	N	NHS Health Check or chronic disease review plus I-CaPP intervention (***N*** = 50)				n	Standard NHS Health Check or chronic disease review (***N*** = 12)			
		Strongly disagree/ disagree n (%)	Neither agree nor disagree n (%)	Agree/ strongly agree n (%)	N/A n(%)		Strongly disagree/ disagree n (%)	Neither agree nor disagree n (%)	Agree/ strongly agree n(%)	N/A n(%)
Increase physical activity	34	1 (3)	7 (21)	24 (71)	2(6)	9	0(0)	3 (33)	6 (67)	0 (0)
Increase fruit and vegetables	34	2 (6)	3 (9)	27 (79)	2 (6)	9	0(0)	3 (33)	6 (67)	0 (0)
Decrease alcohol	34	1 (3)	9 (26)	18 (53)	6 (18)	9	1 (11)	1 (11)	3 (33)	4 (44)
Decrease red meat	35	1 (3)	8 (23)	21 (60)	5 (14)	9	1 (11)	1 (11)	4 (44)	3 (33)
Decrease processed meat	35	1 (3)	6 (17)	22 (63)	6 (17)	9	1 (11)	0 (0)	5 (56)	3 (33)
Stop smoking	34	0 (0)	1 (3)	3 (9)	30 (88)	7	1 (11)	0 (0)	0 (0)	6 (86)
Lose weight	34	0 (0)	3 (9)	25 (74)	6 (18)	9	1 (11)	1(11)	7 (67)	1(11)

**Table 7:** Lifestyle and estimated risk-related outcomes

	**NHS Health Check or chronic disease review plus I-CaPP intervention**				**Standard NHS Health Check or chronic disease review**			
		**Baseline** **(*n*** **= 50)**		**3 month follow-up** **(*n*** **= 30)**		**Baseline** **(*n*** **= 12)**		**3 month follow-up** **(*n*** **= 7)**
	**n**	**Mean (SD)**	**n**	**Change from baseline Mean (95% CI)**	**n**	**Mean (SD)**	**n**	**Change from baseline** **Mean (95% CI)**
**Lifestyle**								
BMI (kg/m^2^)	50	27.5 (5.6)	30	1.14 (−0.46 to 2.74)	12	29.7 (8.0)	7	−0.61 (−2.82 to 1.59)
Alcohol (units/week)	50	9.8 (13.6)	30	−2.8 (−4.9 to −0.66)	12	6.2 (8.7)	7	0 (−1.60 to 1.60)
Physical Activity (hours/week)	50	5.7 (7.7)	30	1.65 (− 0.95 to 4.25))	12	5.1 (8.0)	7	0.21 (−0.91 to 1.34)
Fruit (portions/day)	50	2.1 (1.4)	30	**0.67 (0.1 to 1.23)**	12	2 (0.9)	7	−0.14 (−0.97 to 0.69)
Vegetables (portions/day)	50	2.6 (1.3)	30	0.1 (−0.30 to 0.50)	12	2.1 (0.7)	7	1.00 (0.08 to 1.92)
Red meat (portions/week)	50	2.1 (1.3)	30	**−0.47 (−0.84 to − 0.09)**	12	1.3 (1.1)	7	0.14 (−0.85 to 1.13)
Processed meat (portions/week)	50	1.6 (1.6)	30	**−0.35 (− 0.68 to − 0.02)**	12	1 (0.7)	7	0.43 (− 0.30 to 1.16)
Risk Estimates*								
RRI	50	1.66 (0.9)	30	**−0.34 (− 0.56 to − 1.28)**	12	1.7 (0.8)	7	− 0.25 (− 0.77 to 0.26)
RR	50	1.0 (0.4)	30	−0.005 (− 0.11 to 0.10)	12	1.1 (0.33)	7	−0.07 (− 0.15 to 0.001)
AR	50	3.6 (2.3)	30	0.49 (−0.36 to 1.34)	12	4.4 (2.2)	7	−0.14 (− 0.96 to 0.69)
		**% (95% CI)**		**% (95% CI)**		**% (95% CI)**		**% (95% CI)**
**Smoking status**	50		30		12		7	
Non-smoker		56.0 (41.6 to 69.4)		63.3 (44.0 to 79.2)		33.3 (10.9 to 67.1)		38.6 (4.2 to 78.5)
Ex-smoker		38.0 (25.3 to 52.5)		36.7 (20.8 to 56.0)		58.3 (26.7 to 84.3)		57.1 (15.0 to 90.9)
Current smoker		6.0 (1.9 to 17.6)		0 (0)		8.3 (0.8 to 50.1)		14.3 (1.0 to 74.3)

RRI - Risk relative to an individual of the same age and sex with a recommended lifestyle and RR- Risk relative to an individual of the same age and sex . *All risk estimates represent the 10 year risk fo developing one of the five most common, gender specific, preventable cancers

## Discussion

We have shown that incorporating a theory-based, risk communication-based brief intervention to promote behaviour change for cancer prevention into primary care consultations is feasible and acceptable to both patients and HCPs. In particular, patients receiving the intervention found the cancer risk information and lifestyle advice presented to them understandable, useful and motivating, and HCPs delivered the intervention with high fidelity and felt that it fitted well within their practice. Although this study was not designed to demonstrate efficacy, our findings also suggest that the intervention has the potential to produce behaviour change without increasing worry or anxiety. This pilot study, therefore, provides support for a future randomised controlled trial of an intervention incorporating risk-based cancer information and linked behaviour change advice into primary care.

However, there were a number of challenges that arose during the study and should be taken into account when interpreting the findings of the pilot study and designing future trials and other studies of behaviour change and cancer prevention in primary care. The biggest challenge was recruitment. From the 630 patients invited to receive the intervention within NHS Health Checks, only 34 patients (5.4%) attended. A further 96 (15.2%) attended for an NHS Health Check outside the study. These numbers are substantially lower than the 57.5% uptake of NHS Health Checks for the first three quarters of 2018–19 across our region of recruitment [24]. They are also lower than uptake in a trial of a physical activity intervention nested within NHS Health Checks in the same region in which 27% (*n* = 373/1380) attended an NHS Health Check outside the study and 14.1% (*n* = 194/1380) took part in the study [25]. There are several reasons why our recruitment may have been lower. Firstly, although it was optional, eligible participants may have been put off by the inclusion of video/audio recording of the consultations. This is supported by the similarly low uptake (6%) among participants invited only for a standard NHS Health Check without the cancer intervention. Secondly, in order to enable a researcher to be present to consent participants and set up the recording equipment and to allow for the additional consultation time required for those taking part in the study, clinics were pre-allocated within each practice. Although these were arranged on different days of the week and at different times, this lack of flexibility to arrange an appointment may have acted as a barrier to recruitment, particularly for participants who were employed or who had caring responsibilities [26]. We were unable to collect data on participants who would have liked to take part but were unable to get an appointment. As reported in other studies [25], there was also variation in uptake between practices. This may have been due in part to the different invitation methods used by the practices: telephone invitations to NHS Health Checks have been shown to be more effective in previous studies than letter invitations [27]. The response rate among participants invited to receive the intervention within chronic disease reviews was higher (25%, *n* = 16/65). This may be because these individuals are accustomed to being invited for annual reviews. Both of these potential explanations suggest that the low levels of recruitment reflected aspects of the research design rather the intervention itself. To address these challenges with recruitment, we suggest that the invitations and appointment booking for future interventions are integrated within existing practice systems and letter invitations enhanced with behavioural insights [27] where telephone invitations are not feasible. The need for additional data collection or recording should also be restricted to only that needed to directly assess implementation.

A second challenge was the time needed to deliver the intervention. This had been identified as a potential challenge by HCPs during the development of the intervention [8] and in previous studies exploring the potential for incorporation of cancer prevention within primary care [7, 28, 29] and health promotion activities more generally [30]. In designing the intervention we had, therefore, sought to minimise the contact time required. This included enabling patients to enter the risk factor information prior to the consultation, developing a website that would auto-populate with that information, and providing a leaflet and facility to print a summary that enabled patients to go back to the website after the consultation. These components were all felt by HCPs to be helpful but the mean time to deliver the intervention of 9 min was almost double our target of 5 min, with a range from 3.1 min to 15.5 min. Some of this time was spent checking and amending the information provided by participants prior to the consultation and moving between the electronic healthcare record and the study website. Integrating the risk assessment and communication components of the intervention into the electronic healthcare record may reduce this time and support HCPs [31] and so should therefore be a priority for any future studies.

A third challenge identified by the HCPs was how best to include the discussion about cancer risk and behaviour change within chronic disease reviews. The intervention used in this study had been modelled on the three components of the NHS Health Check (risk assessment, risk communication, risk management). HCPs felt it fitted well within that context but that a clearer introduction may be needed within chronic disease reviews where risk of disease is not discussed as explicitly. Integrating the intervention within the electronic health record may also help with this, particularly if it were incorporated within existing templates.

Despite these challenges, the views of both patients and HCPs on incorporating the intervention within primary care consultations were generally very positive. In particular, the HCPs involved in this study reaffirmed the findings of previous research that has shown how prevention activities are felt to be an important part of their role [7, 28, 29, 32]. The high fidelity of delivery of the intervention also reflects the high levels of engagement among the HCPs and the potential value they saw in the intervention. Unlike HCPs in other studies [28, 30, 33], the HCPs in this study did not appear concerned about a lack of efficacy of the intervention or discuss the need for long term follow-up and referral services. This may have been because the focus of this study was on their role and how the intervention integrated within their practice. Patients also reflected positively on both the delivery and content of the intervention. Few though went back to look at the website after the consultation, questioning the value of that element in future interventions.

These findings must be interpreted in the context of the limitations. A key limitation is the fact that the five general practices included were self-selected and all in one geographical region in the East of England, with four of the five practices in the two least deprived deciles, and all serving a predominantly white population. We cannot therefore comment on whether the challenges with recruitment or the views of HCPs and patients would be different in other regions and among areas with higher deprivation or different ethnic characteristics. The participants themselves were also mostly of low deprivation and well-educated and many were already following a number of the lifestyle recommendations. This mirrors findings for uptake of NHS Health Checks in general [34]. They had also self-selected to attend and so may have been more health aware or concerned about their risk of cancer than the general population and we only collected self-report data on health-related behaviours that could be influenced by social desirability bias. Understanding, if possible, why eligible participants chose not to take part in the study and collecting objective measures of behaviour would be of value in future studies.

## Conclusion

This pilot study of a theory-based brief intervention shows that incorporating discussions about cancer risk and lifestyle advice to promote behaviour change for cancer prevention within primary care consultations is feasible and acceptable to both patients and HCPs. A randomised controlled trial is now needed to assess the effect on health behaviours. When designing that trial, and other prevention activities within primary care, there is a need to consider potential challenges around patient recruitment, the HCP contact time needed for delivery of any interventions, and how best to integrate discussions about cancer risk seamlessly within routine care.

## Supplementary Information

**Additional File 1.** GP practice characteristics.

**Additional File 2.** Healthcare professional training guidance for delivery of the intervention.

**Additional File 3.** Patient interview schedule.

**Additional File 4.** Healthcare professional interview schedule.

**Additional File 5.** Outcome measures.

**Additional File 6.** Questionnaires for intervention group.

**Additional File 7.** Questionnaires for standard NHS Health Check or chronic disease review group.

**Additional File 8.** Psychological outcomes and risk factor awareness within the groups receiving the intervention.

## Data Availability

The datasets used and/or analysed during the current study are available from the Cambridge data repository (10.17863/CAM.63099) on reasonable request. Formal requests for access will be considered through the repository via a data sharing agreement that indicates the criteria for data access and conditions for research use and will incorporate privacy and confidentiality standards to ensure data security.

## Abbreviations

NHS: National Health Service
GP: General Practitioner
CVD: Cardiovascular disease
HCP: Healthcare professional

## References

[1] Cancer Research UK. Cancer statistics. http://www.cancerresearchuk.org/health-professional/cancer-statistics.

[2] Cancer Research UK Statistics on preventable cancers. http://www.cancerresearchuk.org/health-professional/cancer-statistics/risk/preventable-cancers. Accessed 9 Aug 2016.

[3] Public Health England (2015). NHS Health Check Best practice guidance.

[4] French DP, Cameron E, Benton JS, Deaton C, Harvie M (2017). Can communicating personalised disease risk promote healthy behaviour change? A systematic review of systematic reviews. Ann Behav Med.

[5] Usher-Smith J, Silarova B, Sharp SJ, Mills K, Griffin SJ (2018). Effect of interventions incorporating personalised cancer risk information on intentions and behaviour: a systematic review and meta-analysis of randomised controlled trials. BMJ Open.

[6] Ismail H, Atkin K (2015). The NHS health check programme: insights from a qualitative study of patients. Health Expect.

[7] Usher-smith JA, Silarova B, Ward A, Youell J, Muir KR (2017). Incorporating cancer risk information into general practice : a qualitative study using focus groups with healthcare professionals. BJGP.

[8] Mills K, Griffin S, Sutton S, Usher-Smith J (2020). Development and usability testing of a very brief intervention for personalised cancer risk assessment to promote behaviour change in primary care using normalisation process theory. Prim Health Care Res Dev.

[9] Eldridge SM, Lancaster GA, Campbell MJ, Thabane L, Hopewell S, Coleman CL (2016). Defining feasibility and pilot studies in preparation for randomised controlled trials: Development of a conceptual framework. PLoS One.

[10] Public Health England. National General Practice profiles. http://fingertips.phe.org.uk/profile/general-practice. Accessed 15 Oct 2019.

[11] English Indices of Deprivation Data. 2019. http://imd-by-postcode.opendatacommunities.org/imd/2019. Accessed 15 Dec 2019.

[12] Digital NHS (2019). Patients registered at a GP practice September 2019.

[13] Schwartz LM, Woloshin S, Black WC, Welch HG (1997). The role of numeracy in understanding the benefit of screening mammography. Ann Intern Med.

[14] Crockett RA, Weinman J, Hankins M, Marteau T (2009). Time orientation and health-related behaviour: measurement in general population samples. Psychol Health.

[15] Ferrer RA, Klein WMP, Persoskie A, Avishai-Yitshak A, Sheeran P (2016). The tripartite model of risk perception (TRIRISK): distinguishing deliberative, affective, and experiential components of perceived risk. Ann Behav Med.

[16] Stubbings S, Robb K, Waller J, Ramirez A, Austoker J, Macleod U (2009). Development of a measurement tool to assess public awareness of cancer. Br J Cancer.

[17] Lerman C, Kash K, Stefanek M. Younger women at increased risk for breast cancer: perceived risk, psychological well-being, and surveillance behavior. J Natl Cancer Inst Monogr. 1994;16:171–6. 7999461

[18] Lerman C, Trock B, Rimer BK, Boyce A, Jepson C, Engstrom PF (1991). Psychological and behavioral implications of abnormal mammograms. Ann Intern Med.

[19] Marteau TM, Bekker H (1992). The development of a six-item short-form of the state scale of the Spielberger state-trait anxiety inventory (STAI). Br J Clin Psychol.

[20] Rippetoe PA, Rogers RW (1987). Effects of components of protection-motivation theory on adaptive and maladaptive coping with a health threat. J Pers Soc Psychol.

[21] Godino JG, van Sluijs EMF, Marteau TM, Sutton S, Sharp SJ, Griffin SJ (2012). Effect of communicating genetic and phenotypic risk for type 2 diabetes in combination with lifestyle advice on objectively measured physical activity: protocol of a randomised controlled trial. BMC Public Health.

[22] Sanderson SC, Persky S, Michie S (2010). Psychological and behavioral responses to genetic test results indicating increased risk of obesity: does the causal pathway from gene to obesity matter?. Public Health Genomics.

[23] Braun V, Clarke V (2006). Using thematic analysis in psychology. Qual Res Psychol.

[24] Public Health England (2019). NHS Health Checks.

[25] Attwood S, Morton K, Sutton S. Exploring equity in uptake of the NHS Health Check and a nested physical activity intervention trial. J Public Health (Oxf). 2016;38(3):560–8 10.1093/pubmed/fdv070 PMC5072157 26036701

[26] Harte E, Maclure C, Martin A, Saunders CL, Meads C, Walter FM, et al. Reasons why people do not attend NHS Health Checks: A systematic review and qualitative synthesis. Br J Gen Pract. 2018;68(666):e28–35 10.3399/bjgp17X693929 PMC5737317 29203682

[27] Bunten A, Porter L, Gold N, Bogle V (2020). A systematic review of factors influencing nhs health check uptake: invitation methods, patient characteristics, and the impact of interventions. BMC Public Health.

[28] McIlfatrick S, Keeney S, McKenna H, McCarley N, McIlwee G (2014). Exploring the actual and potential role of the primary care nurse in the prevention of cancer: a mixed methods study. Eur J Cancer Care.

[29] McIlfatrick S, Keeney S, McKenna H, McCarley N, McElwee G (2013). Investigating the role of the general practitioner in cancer prevention: a mixed methods study. BMC Fam Pr.

[30] Geense WW, van de Glind IM, Visscher TLS, van Achterberg T (2013). Barriers, facilitators and attitudes influencing health promotion activities in general practice: an explorative pilot study. BMC Fam Pract.

[31] Dickfos M, King D, Parekh S, Boyle FM, Vandelanotte C (2015). General practitioners’ perceptions of and involvement in health behaviour change: can computer-tailored interventions help?. Prim Health Care Res Dev.

[32] Brotons C, Björkelund C, Bulc M, Ciurana R, Godycki-Cwirko M, Jurgova E (2005). Prevention and health promotion in clinical practice: the views of general practitioners in Europe. Prev Med (Baltim).

[33] Schütze H, Rix EF, Laws RA, Passey M, Fanaian M, Harris MF (2012). How feasible are lifestyle modification programs for disease prevention in general practice?. Aust J Prim Health.

[34] Martin A, Saunders CL, Harte E, Griffin SJ, MacLure C, Mant J (2018). Delivery and impact of the NHS health check in the first 8 years: a systematic review. Br J Gen Pract.

