# Supplementary material for: Incorporating a brief intervention for personalised cancer risk assessment to promote behaviour change into primary care: a multi-methods pilot study
Source: BMC Public Health. 2021 Jan 23;21:205. doi: 10.1186/s12889-021-10210-3 (PMC7824918; doi:10.1186/s12889-021-10210-3)
Supplement: Supplementary file 1 — Additional File 1. GP practice characteristics. [file 12889_2021_10210_MOESM1_ESM.pdf]

Additional File 1. GP practice characteristics

NHS Health Checks				
**GP practice**	**Registered patients (n)**	**Deprivation index decile (1 most deprived-10 least deprived)**	**HCP delivering intervention (n)**	**Methods of invitation**
A	10438	9	1	Letter
B	12567	6	2	Letter, follow-up telephone call
C	7893	10	2	Letter
D	12057	9	2	Letter, follow-up telephone call
**Total**	**42955**		**7**	
**Chronic disease reviews**				
**GP practice**	**Registered patients (n)**	**Deprivation index decile (1 most deprived-10 least deprived)**	**HCP delivering intervention (n)**	**Methods of invitation**
D (Diabetes)	12057	9	2	Letter
E (Diabetes)	8407	9	2	Letter, follow-up telephone call, text message
E (Asthma)				
**Total**	**20464**		**4**	
