# Supplementary material for: Incorporating a brief intervention for personalised cancer risk assessment to promote behaviour change into primary care: a multi-methods pilot study
Source: BMC Public Health. 2021 Jan 23;21:205. doi: 10.1186/s12889-021-10210-3 (PMC7824918; doi:10.1186/s12889-021-10210-3)
Supplement: Supplementary file 2 — Additional File 2. Healthcare professional training guidance for delivery of the intervention. [file 12889_2021_10210_MOESM2_ESM.pdf]

UNIVERSITY OF CAMBRIDGE

Funded by CANCER RESEARCH UK

I-CaPP

Healthcare professional pathway in the preparation and delivery of the I-CaPP study consultations

Prior to the start of the I-CaPP session, the study researcher will arrive to set-up the recording equipment and will be on hand to provide technical support and any guidance needed during the session.

1. You will first complete a consent form with the study researcher before the arrival of the first patient.

Each patient will arrive and see the study researcher in the waiting room before the start of the consultation for baseline questionnaire completion, if not already completed at home. The study researcher will provide information, answer questions about the study and obtain written informed consent from each participant. The last questions completed by the patient are for the risk calculation. These include: gender; age; body weight; height; smoking status; number of portions of fruit and vegetables eaten on a typical day; number of portions of red and processed meat eaten in a typical week; hours of moderate physical activity per week, number of units of alcohol per week.

On completion of the consent form each patient will be handed a slip to bring into their consultation. The slip will indicate if the patient is happy for one of the following:

video recording	audio recording	or no recording.
✓	✓	X

The participant ID will be written at the bottom of this slip. This participant ID will be used to access and calculate the patient's risk on the website.

2. As the patient enters the consultation room, turn on the appropriate recorder.
3. Complete the NHS Health Check as you would normally, including providing the patient with their QRisk result.
4. Next, share with the patient the inclusion of cancer information to the NHS Health Check programme is being trialled in the I-CaPP study. This is taking place in a small group of GP practices currently, but if helpful to people we aim to roll this out across the country.
5. Open browser (preferably google chrome) and go to website address #####
6. Enter participant ID.
7. Once you click "next" the values entered for each question of the risk calculation will be displayed as a table, show below.

Cancer Risk

Estimating the risk of people of your age and sex and with your lifestyle developing one of the five most common preventable cancers over the next 10 years. For men these are lung, bowel, bladder, kidney and oesophageal cancer and for women these are breast, lung, bowel, womb and kidney cancer.

	**Your values now**
Height, (in m)	1.8
Weight, (in kg)	50
BMI, (automatically calculated)	15.4
Smoking status	Smoker
Fruit, (portions per day)	2
Vegetables, (portions per day)	2
Red meat, (portions per week)	2
Processed meat, (portions per week)	6
Alcohol, (units per week)	2
Physical activity, (hours per week)	2

Calculate risk

8. If any of the values need to be changed (this may be relevant if the patient has already mentioned certain lifestyle factors as part of the health check and are different to what they entered by themselves in the waiting room) this can be done now before you select the button “calculate risk”.
   The next screen will show you the risk presentation, as shown below.
9. Please explain to the patient that this is the estimated 10-year cancer risk of someone like them when compared to people of the same age and gender who follow all of the recommended lifestyle guidelines. The recommended guidelines include:
   a. BMI within the normal range (18.5-24.9)
   b. No smoking
   c. At least 2.5 portions of fruit per day
   d. At least 2.5 portions of vegetables per day
   e. No red meat each week
   f. No processed meat each week
   g. No units of alcohol each week
   h. At least 2.5 hours of physical activity each week

These are also displayed at the top of the webpage as a reference.

10. Show and describe the risk to the patient. Please describe the risk level shown on the left, highlighting the colour of the risk level and that green is the lowest risk, and red puts them at the highest risk. The axis will indicate how many times higher their current risk level is compared to the recommended guidelines.
    If it helps them to understand the risk level or they would like to see it, the percentage of this risk can be displayed by clicking on the button at the bottom of the screen to show this . To help the patient understand this figure it may be useful to explain that this percentage means that if there were 100 people of the same age and gender and with the same lifestyle as them over the next 10 years the estimated percentage shown would develop one of the five cancers.
11. Next with the links on the right hand side of this page, discuss with the patient, which of the lifestyle factors they would like to consider setting new goals for to help to reduce their current estimated risk. Promote behaviour change emphasising the link between CVD and Cancer.

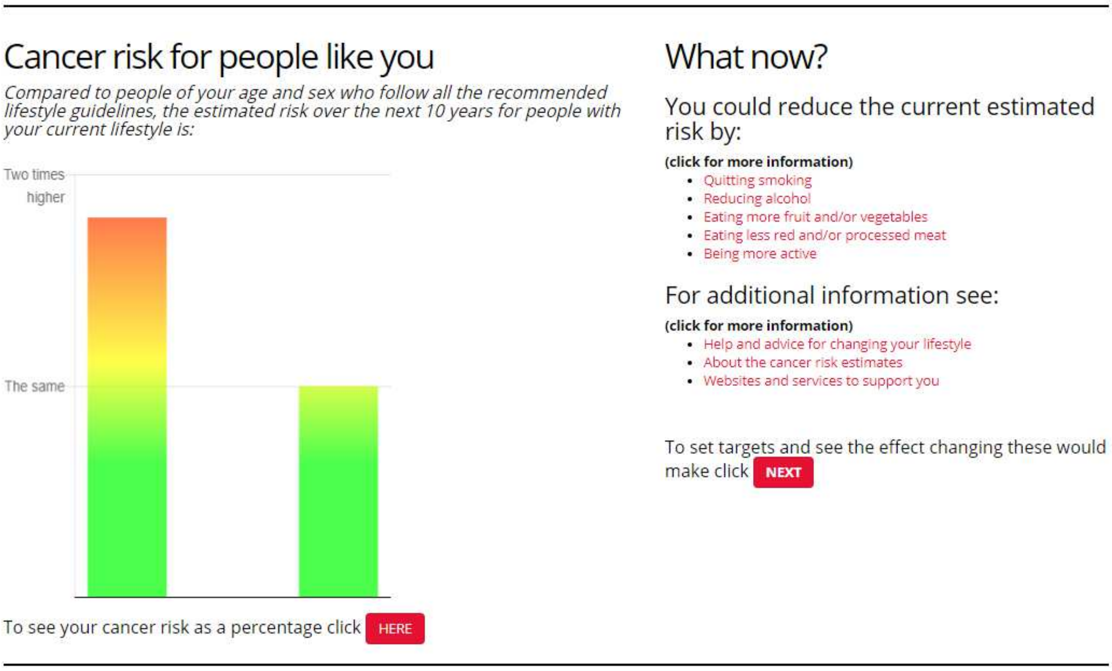

12. Key messages to get across:
    a. Lifestyle factors that increase risk of cardiovascular disease also increase the risk of developing cancer,
    b. Adopting a healthier lifestyle people can reduce the risk of developing both cardiovascular disease and cancer.

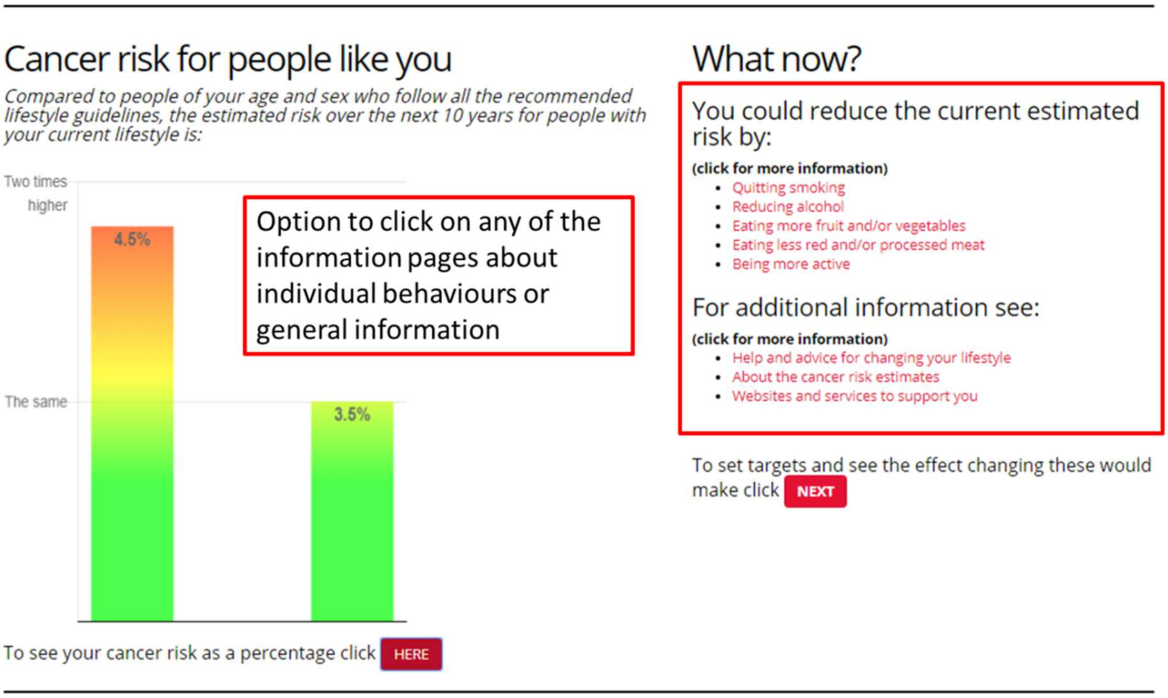

13. By clicking on any of these links a new webpage will open to show specific information to inform and guide the patient to set new targets. Here is any example of one of the lifestyle pages.

Eating more fruit & vegetables

Fruit and vegetables contain lots of important nutrients, which are vital for a healthy diet. They are low in calories and are an excellent source of fibre so can help you to keep at a healthy weight. Eating more fruit and vegetables can particularly help to reduce your risk of mouth, throat, lung and bowel cancers.

Experts suggest we eat 5 or more portions of fruit and vegetables each day. A portion is the same as a **medium-sized apple, a handful or small fruit, or 3 tablespoons of vegetables.**

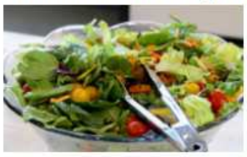

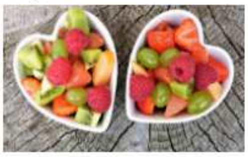

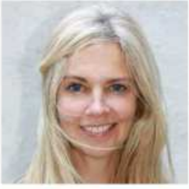

" I try to eat 5 portions of colourful fruit and vegetables each day"

What changes could you make to include more fruit and vegetables in your diet?

Some ways that have worked for others include:

- Add fruit to your breakfast cereal
- Chop raw vegetables carrots, peppers, cherry tomatoes, cucumber sticks to snack on during the day
- Try adding a side salad to your lunch/dinner
- Include an extra portion of vegetables to your dinner
- Have a piece of fruit as your dessert each day

Use this space to write down your goal and plan how and when you will do your new action

Print Information

Return to my risk

14. There is a box on this page where the patient's goal can be entered, if helpful.
15. Next, return to the risk page by selecting "return to my risk".
16. On returning to this page, select the button highlighted below to "set targets to see the effect of changing these would make".

Cancer risk for people like you

Compared to people of your age and sex who follow all the recommended lifestyle guidelines, the estimated risk over the next 10 years for people with your current lifestyle is:

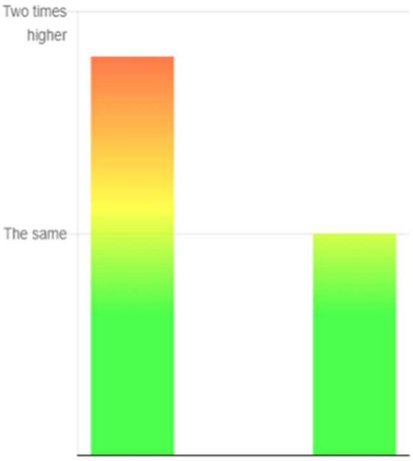

To see your cancer risk as a percentage click HERE

What now?

You could reduce the current estimated risk by:

(click for more information)

- Quitting smoking
- Reducing alcohol
- Eating more fruit and/or vegetables
- Eating less red and/or processed meat
- Being more active

For additional information see:

(click for more information)

- Help and advice for changing your lifestyle
- About the cancer risk estimates
- Websites and services to support you

To set targets and see the effect changing these would make click NEXT

17. This will take you back to the top of the page where you will be able to see the current values for each of the risk factors and enter new ones with the patient. The recommended values are also here to act as a guide.

Cancer Risk

Estimating the risk of people of your age and sex and with your lifestyle developing one of the five most common preventable cancers over the next 10 years. For men these are lung, bowel, bladder, kidney and oesophageal cancer and for women these are breast, lung, bowel, womb and kidney cancer.

	Your values now	Your target values	Your recommended values
Height, (in m)	1.8	1.8	
Weight, (in kg)	50	50	
BMI, (automatically calculated)	15.4	15.4	Less than 25
Smoking status	Smoker	Smoker	Non-Smoker
Fruit, (portions per day)	2	2	At least 2.5
Vegetables, (portions per day)	2	2	At least 2.5
Red meat, (portions per week)	2	2	Ideally 0
Processed meat, (portions per week)	6	6	Ideally 0
Alcohol, (units per week)	2	2	Ideally 0
Physical activity, (hours per week)	2	2	At least 2.5

Calculate target risk

18. Once the new target values have been entered, select “calculate target risk”. This will then display the new risk estimate alongside the original risk calculated and the lowest possible risk when following the recommend lifestyle guidance.
19. Describe to the patient the reduction in risk shown, emphasising the change in colour of the graph if appropriate. Again, the percentages can be displayed if this aids patient understanding.

Cancer risk for people like you

Compared to people of your age and sex who follow all the recommended lifestyle guidelines, the estimated risk over the next 10 years for people with your current lifestyle is:

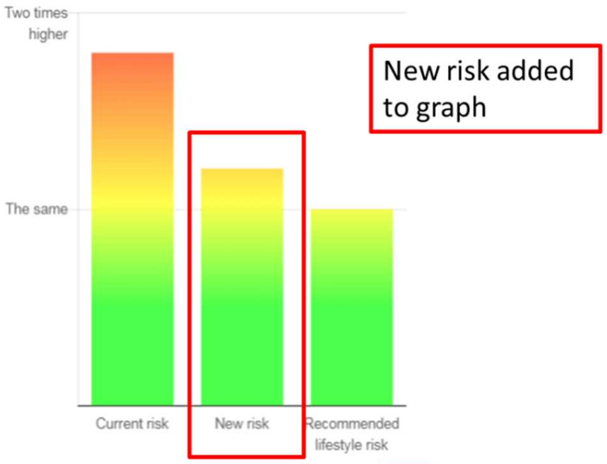

To see your cancer risk as a percentage click HERE

What now?

You could reduce the current estimated risk by:

(click for more information)

- Quitting smoking
- Reducing alcohol
- Eating more fruit and/or vegetables
- Eating less red and/or processed meat
- Being more active

For additional information see:

(click for more information)

- Help and advice for changing your lifestyle
- About the cancer risk estimates
- Websites and services to support you

To set targets and see the effect changing these would make click HERE

If the patient would like to see how other changes could influence their estimated risk there is the option to do so by selecting “set targets and see the effect change these would make”. This can be updated as many times as needed.

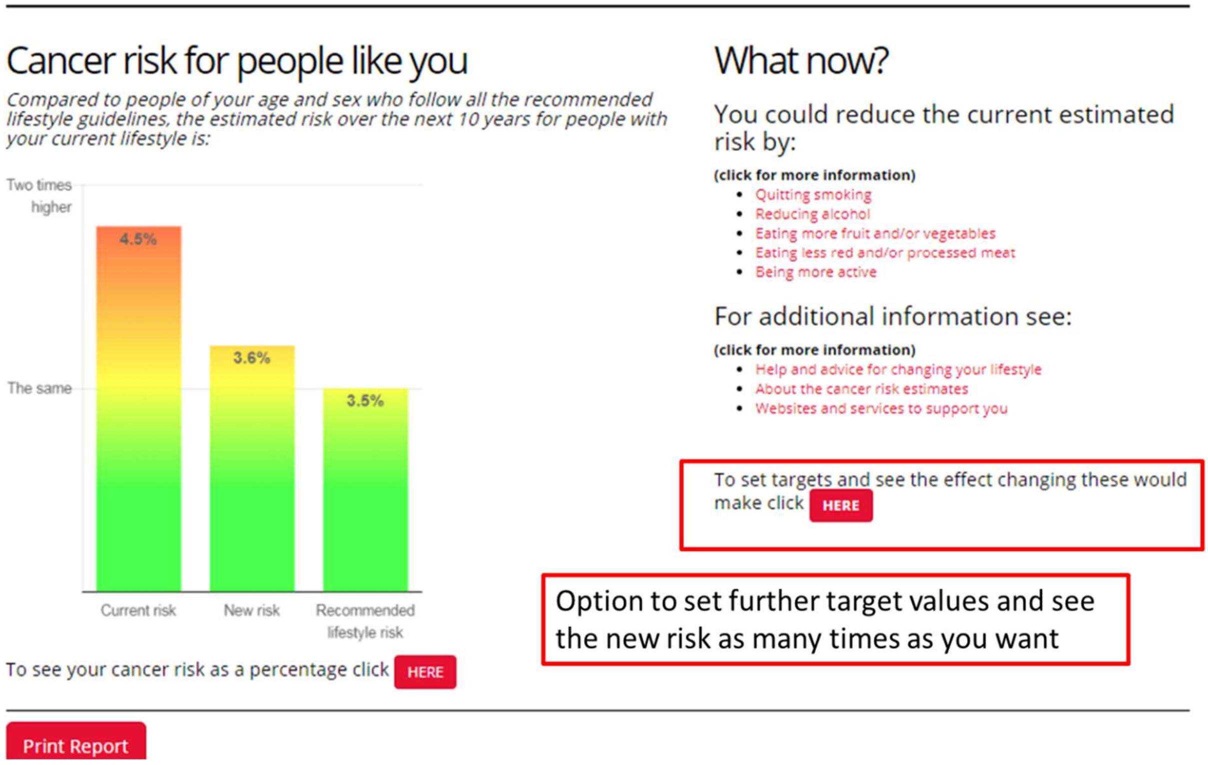

20. On completion of the discussion about risk, please signpost the participants to additional information that can obtained. These are available by clicking on the links or at the back of the paper leaflet. Also, advise patients that Cancer Research UK provides lots of resources on their website.
21. Next, print the risk page.

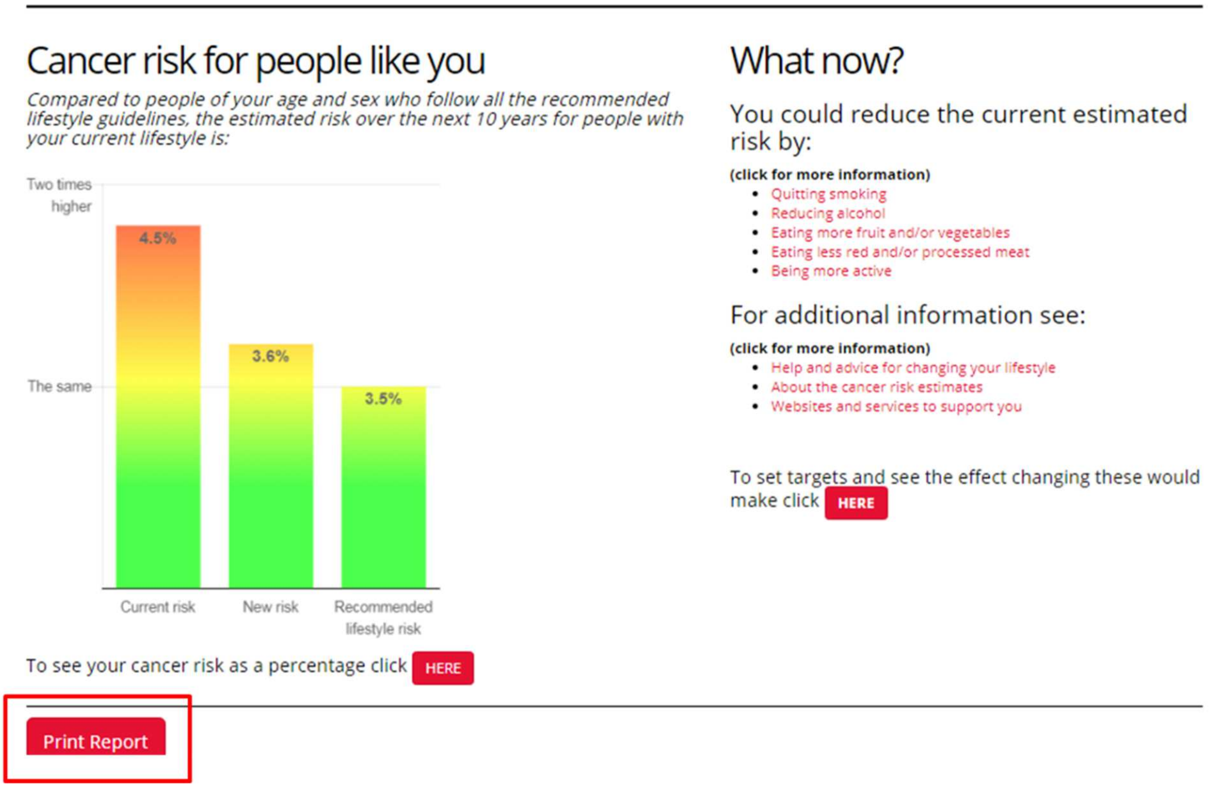

22. On printing, at the bottom of the second page, the participant ID and the website address is printed. Please inform the patient that they are able to revisit the website to for further information, look back at their risk and set new targets.

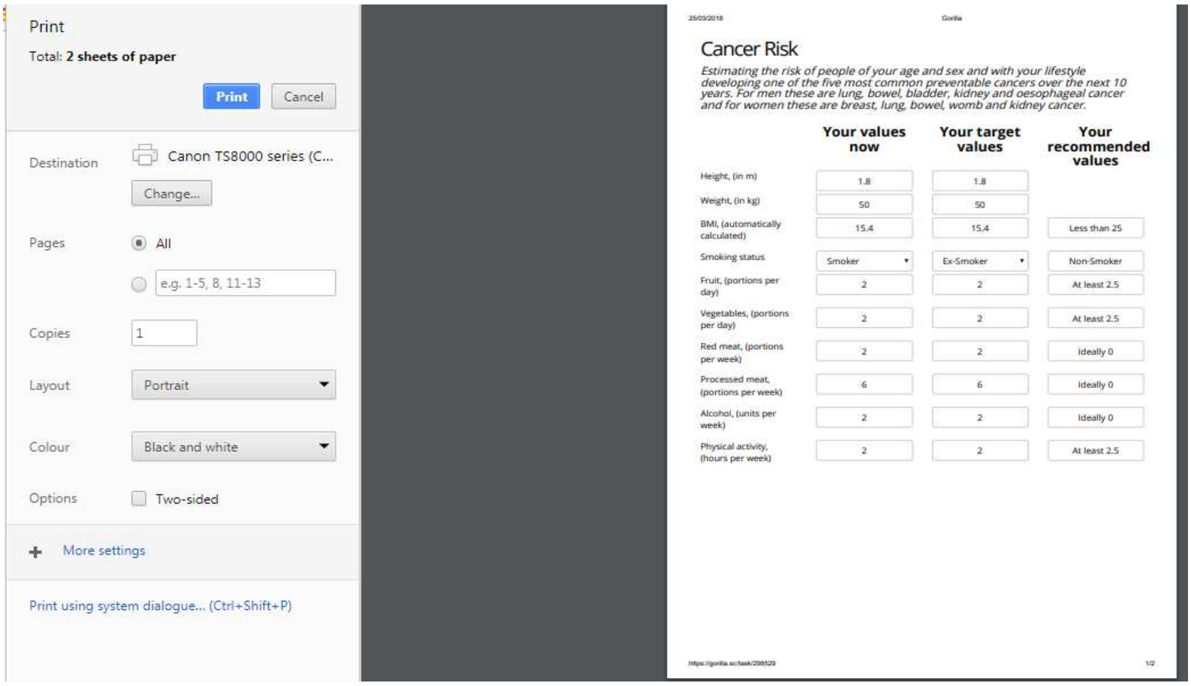

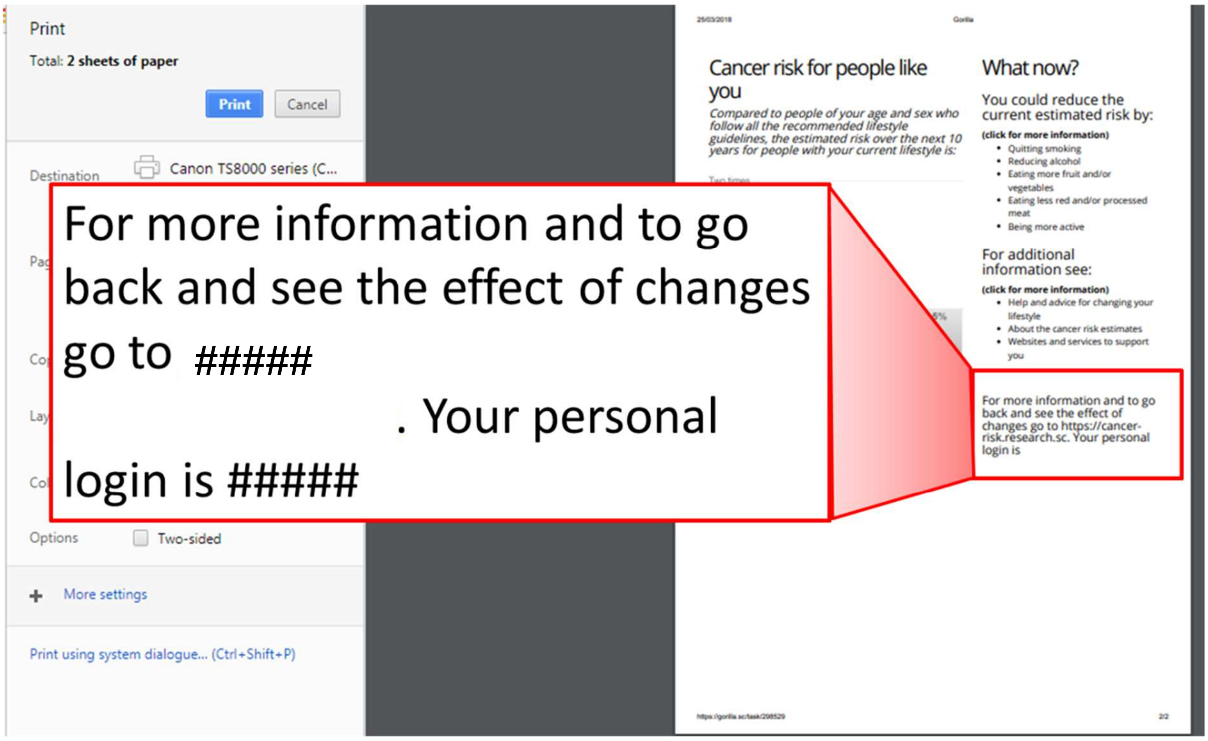

23. Provide patients with the opportunity to ask questions about their risk and alleviate any concerns. Answers to commonly asked questions can be found in the lifestyle leaflet, shown below. Signpost them to see their GP if they would like to discuss their concerns further.

Frequently asked questions

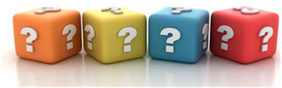

What if I am already living a healthy lifestyle?
You may find after reading this leaflet that you are already making healthy choices but there could still be opportunities to do more. Perhaps consider increasing the amount of time you spend doing physical activity each week and aim to increase this over time.

Is it too late in my life to start making changes?
Definitely not. Any small changes you can make can help to reduce your risk of developing cancer in the future.

Where can I get more information on living a healthy lifestyle?
There are websites on the following page that you can use to find further information. If you would like to speak to someone there is also information here of local services you can access to get additional support and advice.

Where do the estimates of cancer risk come from?
The estimates are calculated based on data from lots of studies which have looked at how lifestyle affects risk of cancer.

How accurate are the estimates?
We have tested the estimates within a population of over 20,000 people living in the East of England and they apply well to that group. However, they do not apply perfectly to all individuals. A high risk estimate does not mean that you are destined to get cancer and a low risk estimate does not mean that you are not going to get cancer. There are also other factors, including other diseases or medications that you take, that can affect the risk of developing cancer.

Why were these five cancers chosen?
There are more than 200 different types of cancer. In order to show the effect of lifestyle we have included the five most common ones that are affected by lifestyle. Adopting a healthy lifestyle will also reduce the risk of developing other types of cancer.

Who can I talk to about my cancer risk?
If you would like to talk to someone about your future risk of cancer please contact your GP to make an appointment.

24. Please give the patient the print out of the risk presentation, a paper copy of the leaflet and also the return the slip they gave you at the start of the consultation. *They will now re-consent for the recording with the study researcher before leaving the practice.*

25. Please turn off the recorder once they have left the room.

At the end of the I-CaPP session, the researcher will ask you to re-consent for the recording of each of the consultations completed.
