# Supplementary material for: Incorporating a brief intervention for personalised cancer risk assessment to promote behaviour change into primary care: a multi-methods pilot study
Source: BMC Public Health. 2021 Jan 23;21:205. doi: 10.1186/s12889-021-10210-3 (PMC7824918; doi:10.1186/s12889-021-10210-3)
Supplement: Supplementary file 3 — Additional File 3. Patient interview schedule. [file 12889_2021_10210_MOESM3_ESM.pdf]

I-CaPP Programme- Pilot Study

Exploring communication of disease risk in primary care
I-CaPP intervention group interview schedule

Introduction

The purpose of this part of the study is to find out about your views on receiving information about risk of cancer in the consultation you had with your healthcare professional. Everything that you say here will be kept confidential, and neither your name nor any other identifying information will be used in any report coming from this research.

Confirm understanding of study, participation & recording, confidentiality issues & consent

1. May I ask you first, at the time when you were invited to take part in this study, what did you know or think about your risk of developing cancer? *[Prompts: What was this perception based on? Is it for cancer in general or specific cancers? Have you discussed your risk with a GP or another healthcare professional before?]*

Immediate reaction to risk information

So hopefully you will remember that in the consultation the healthcare professional they showed you your estimated risk of developing one of the five most common preventable cancers.

2. Do you remember the information about your risk that you were given?

3. Do you remember what your first reaction was when you were given that? *[Prompts: surprised, worried/ relieved]*

4. Different people understand different things when they see information of this kind - what did you understand from it? *[Prompts: What do the results mean? Do you think this is a high score, a low score, not high and not low?]*

5. How does that compare to what you thought about your risk of developing cancer beforehand?

Hand participant a copy of the printed information they were given.

6. What did you think about the way the risk was presented? *[Prompts: Did you like the graph/text, was anything unclear?]*

7. Is there anything that you think might have made it clearer or helped your understanding of the risk scores? [*Prompts: layout of the webpage, longer discussion*

with the nurse]

8. How do you think being given information about risk of cancer compares to being given information for other conditions such as heart attack and stroke or diabetes?

9. Do you remember the information about your risk of cardiovascular disease that you were given?

10. Do you remember what your first reaction was when you were given that? *[Prompts: surprised, worried/ relieved]*

11. Different people understand different things when they see information of this kind - what did you understand from it? *[Prompts: What do the results mean? Do you think this is a high score, a low score, not high and not low?]*

12. How does that compare to what you thought about your risk of developing cardiovascular disease beforehand?

General views on website and lifestyle advice
Thinking now about the lifestyle advice in the leaflet and available on the website.

13. Did you learn anything new? (*If yes*) What did you learn? Was anything a surprise?

14. How is this different from what you thought about your risk before you received this information?

15. What did you think about the lifestyle information? *[Prompts: content/format/length – too detailed/too simple?]*

16. Did you go back to the website after the consultation? Did you try looking at what would happen if you changed your lifestyle?

17. Did you read the leaflet?

Potential impact

18. Do you think seeing your risk of cancer and the lifestyle information has made any difference to you? Do you think you will change anything in your lifestyle as a result of seeing your risk? *[Prompts: Why?]*

19. Did you discuss your risk with anyone else? [Prompts: family members, friends/ visited healthcare professional?]

Overall views

20. Do you think this information should be available to everyone?

21. If it was not part of a study, do you think the NHS health check was the right place to be given the information or would you have preferred it in online or in other consultations/from other professionals? [Prompts: Why?]

22. Would you recommend to your friends and family to find out about their risk of cancer?

Views of the study process

23. Do you have any comments on the questionnaires you completed or your overall involvement in the study?

Any other questions and thank you

24. I have no more questions for you. Do you have anything else you would like to add?

25. Do you have any questions for me?

26. Thank you very much for taking part
