# Supplementary material for: Incorporating a brief intervention for personalised cancer risk assessment to promote behaviour change into primary care: a multi-methods pilot study
Source: BMC Public Health. 2021 Jan 23;21:205. doi: 10.1186/s12889-021-10210-3 (PMC7824918; doi:10.1186/s12889-021-10210-3)
Supplement: Supplementary file 4 — Additional File 4. Healthcare professional interview schedule. [file 12889_2021_10210_MOESM4_ESM.pdf]

I-CaPP Programme- Pilot Study

Exploring communication of disease risk in primary care
Healthcare professional interview schedule

1. Welcome and introduction including explanation that we are studying the implementation of a new risk based cancer intervention during NHS Health Checks

2. Confirm understanding of study, participation & recording, confidentiality issues & consent

3. How did you find using the tool? Were there instances when it was useful/helpful and others when you found it less so? [Particular patient groups you think it has the potential to work well with/wouldn't work with?]

4. Thinking about the risk information on the website, what do you think of it? *[Prompts: Do they like it? Not like it? Why/why not? Prompt for ease of use, functionality, interface and graphics. How does it compare to other risk tools?]*

5. What did you think about the lifestyle advice on the website and in the leaflet?

6. How did you feel using the website to give patients their risk of cancer?

7. Did you feel comfortable discussing risk of cancer with patients?

8. Did you use the website to demonstrate how changing lifestyle influences risk with patients in consultations? *[Prompts: Was that helpful?]*

9. Do you think including discussions about cancer changed the consultation? *[Prompts: Any impact on referrals or management?]*

10. What do you see as the advantages and disadvantages of using the tool? *[Prompts: in process, communication, to colleagues and the practice, on lifestyle changes; advantages, clinical tool and framework for assessment; disadvantages, time, management of patient based on risk unclear, medico-legal concerns]*

11. Could you see yourself using it routinely in NHS Health Checks? *[Prompts: If not, why not? What resources would they need?]*

12. What about other consultations?

[Prompts: e.g. chronic disease reviews, routine consultations; Are there some consultations where you think it would fit better than others? What would it take for you to want to use the tool in routine practice?]

13. Did you feel you had enough training on using the intervention?

14. What improvements would you make to the website and/or leaflet to make the intervention more useful?
    [*Prompts*: Changes to interface, risk outputs, other functionality, electronic prompts]

15. Have you discussed the intervention with others in your practice?

16. If yes, what is the general feeling among staff members about the tool?
    [Prompts: different opinions of nurses and GPs, would they recommend it to colleagues?]

17. Would you support the introduction of the intervention into NHS Health Checks +/- other consultations? *[Prompts: If not, why not?]*

18. Do you think healthcare assistants and others in the practice could deliver the intervention?

19. Is there anything else you'd like to discuss?

20. Thanks, confirmation of consent and confidentiality
