# Supplementary material for: Incorporating a brief intervention for personalised cancer risk assessment to promote behaviour change into primary care: a multi-methods pilot study
Source: BMC Public Health. 2021 Jan 23;21:205. doi: 10.1186/s12889-021-10210-3 (PMC7824918; doi:10.1186/s12889-021-10210-3)
Supplement: Supplementary file 5 — Additional File 5. Outcome measures. [file 12889_2021_10210_MOESM5_ESM.pdf]

Additional File 5. Outcome measures

Measure		Data collection point					
		Baseline		Immediately post-intervention		3-months post-intervention	
		Intervention	Standard consultation	Intervention	Standard consultation	Intervention	Standard consultation
Demographics	*Details*						
Age	Years	✓	✓	-	-	-	-
Gender	Male/Female	✓	✓	-	-	-	-
Ethnicity	White / Mixed/Multiple ethnic group / Asian/Asian British / Black/ African/Caribbean / Other	✓	✓	-	-	-	-
Family history of cancer	Assessed by one question “Have your parents or any brothers or sisters ever had cancer?”	✓	-	-	-	-	-
Family history of CVD	Assessed by one question “Have your parents or any brothers or sisters ever had cardiovascular disease?”	✓	✓	-	-	-	-
Highest education level	No formal education / Primary education / Secondary education / University education	✓	✓	-	-	-	-
Postcode	Used to calculate Index of Multiple Deprivation	✓	✓	-	-	-	-
Lifestyle							
Self-reported weight	-	✓	✓	-	-	✓	✓
Self-reported height	-	✓	✓	-	-	✓	✓
Smoking status	current/ex-smoker or never smoked	✓	✓	-	-	✓	✓
Alcohol consumption	units/week	✓	✓	-	-	✓	✓
Physical activity	hours/week	✓	✓	-	-	✓	✓
Fruit consumption	portions/day	✓	✓	-	-	✓	✓
Vegetable consumption	portions/day	✓	✓	-	-	✓	✓
Red meat consumption	portions/week	✓	✓	-	-	✓	✓
Processed meat consumption	portions/week	✓	✓	-	-	✓	✓
Other outcome measures							
Awareness of cancer risk factors	Questions 6 and 8 from the CR-UK Perceptions of risk survey (1)	✓	-	✓	-	✓	
Cancer risk perception	Absolute and comparative perception of risk using both a continuous and categorical measure as in previous studies(2) adapted for cancer (e.g. “On a scale from 0 to 100%, how would you rate the probability that you will develop one of these five cancers in the next 10 years?”, and “How do you think your chance of developing one of these five cancers in the next 10 years compares to the average person of your sex and age?”)	✓	-	✓	-	✓	-
CVD risk perception	Absolute and comparative perception of risk using both a continuous and categorical measure as for cancer risk	✓	✓	✓	✓	✓	✓
Cancer worry	The Lerman Cancer Worry Scale(3)	✓	-	-	-	✓	-

Cancer anxiety	Short form of the Spielberger State Trait Anxiety Inventory (STAI)(4)	✓	-	-	-	✓	-
Maladaptive coping	Assessed using three questions measuring avoidance, fatalism and hopelessness as used in previous research(5): “I try not to think about the possibility of developing cancer.” [*Avoidance*] “If you are destined to develop cancer you will, there is really very little you can do about it.” [*Fatalism*] “Given what I know about cancer, I sometimes feel it is almost useless to try to stay healthy.” [*Hopelessness*]	✓	-	✓	-	✓	-
Self-efficacy	Assessed using 3 Likert items for each of physical activity and fruit and vegetables evaluated on a 5-point response scale ranging from “strongly disagree” to “strongly agree” as used in previous research(6) adapted for use in the context of cancer (e.g. “I would like to be physically active but I don’t know if I can.”, “I feel confident in my ability to be active at a moderate intensity for at least 30 minutes per day on at least 5 days a week.”, and “I am confident that I could be physically active if I wanted to.”.	-	-	✓	-	-	-
Response efficacy	Assessed using 3 Likert items for each of physical activity and fruit and vegetables evaluated on a 5-point response scale ranging from “strongly disagree” to “strongly agree” as used in previous research(6) adapted for use in the context of cancer (e.g. ‘Eating a healthy diet is effective in preventing cancer”, “Eating a healthy diet works in preventing cancer”, and “If I eat a healthy diet, I am less likely to develop cancer”.	-	-	✓	-	-	-
Intention to change behaviour	Overall intention assessed using four questions: “I am determined to do everything I can to avoid getting cancer in the future.” “I am committed to engaging in behaviours that protect me against getting cancer in the future.” “I fully intend to have a lifestyle that will prevent me from getting cancer in the future.” “I will try to do all I can to avoid getting cancer in the future.” evaluated on a 7-point response scale from “Strongly agree” to “Strongly disagree” and treated as a continuous variable. Specific intention assessed using questions for each behaviour e.g. “I intend to be more physically active in the next 3 months”) evaluated on a 7-point response scale from “Strongly agree” to “Strongly disagree”, again each treated as continuous variables.	-	-	✓	✓	-	-
Potential mediators and moderators							
Numeracy	The 3-item Schwartz scale(7)	✓	✓	-	-	-	-
Time orientation	The brief nine item form of the Zimbardo Time Perspective Inventory (ZTPI-R)(8)	✓	✓	-	-	-	-
Self-rated health	Assessed with one question “How would you rate your general health?” on a 5-point scale from “Very good” to “Poor”	✓	✓	-	-	-	-

Previous risk information of developing cancer	Assessed with one question "Have you ever received information on your risk of developing cancer?"	✓					
Understanding and acceptability of the intervention				✓			

References

1. Cancer Research UK. Perceptions of Risk Survey 2008 : Key Findings. 2008; Available from: www.cancerresearchuk.org/sites/default/files/perceptions_of_risk_survey.pdf
2. Godino JG, van Sluijs EMF, Sutton S, Griffin SJ. Understanding perceived risk of type 2 diabetes in healthy middle-aged adults: a cross-sectional study of associations with modelled risk, clinical risk factors, and psychological factors. Diabetes Res Clin Pract. 2014;106(3):412–9.
3. Lerman C, Trock B, Rimer BK, Jepson C, Brody D, Boyce A. Psychological side effects of breast cancer screening. Heal Psychol. 1991;10(4):259–67.
4. Marteau TM, Bekker H. The development of a six-item short-form of the state scale of the Spielberger State-Trait Anxiety Inventory (STAI). Br J Clin Psychol. 1992 Sep;31 (Pt 3):301–6.
5. Rippetoe P a, Rogers RW. Effects of components of protection-motivation theory on adaptive and maladaptive coping with a health threat. J Pers Soc Psychol. 1987;52(3):596–604.
6. Sanderson SC, Persky S, Michie S. Psychological and Behavioral Responses to Genetic Test Results Indicating Increased Risk of Obesity: Does the Causal Pathway from Gene to Obesity Matter? Public Health Genomics. 2010;13(1):34–47.
7. Schwartz LM, Woloshin S, Black WC, Welch HG. The role of numeracy in understanding the benefit of screening mammography. Ann Intern Med. 1997;127(11):966–72.
8. Crockett RA, Weinman J, Hankins M, Marteau T. Time orientation and health-related behaviour: Measurement in general population samples. Psychol Heal. 2009;24(3):333–50.
