# Supplementary material for: Incorporating a brief intervention for personalised cancer risk assessment to promote behaviour change into primary care: a multi-methods pilot study
Source: BMC Public Health. 2021 Jan 23;21:205. doi: 10.1186/s12889-021-10210-3 (PMC7824918; doi:10.1186/s12889-021-10210-3)
Supplement: Supplementary file 6 — Additional File 6. Questionnaires for intervention group. [file 12889_2021_10210_MOESM6_ESM.pdf]

I-CaPP Programme- Pilot Study

Exploring communication of disease risk in primary care

Questionnaires for intervention group

[Note that this content was delivered online so the formatting looked different to in this document and participants did not see the subheadings highlighted in blue. The participants also had clear instructions on how to respond to each question depending on the format e.g. radio buttons/drop-down lists]

Baseline Questionnaire

Thank you for agreeing to complete this questionnaire. Please answer every question. If you are uncertain about how to answer a question, then please select the closest option. There are no right or wrong answers.

About you and your health

How would you rate your general health?

- Very good
- Quite good
- Neither good nor poor
- Quite poor
- Poor

Have your parents or any brothers or sisters ever had cancer?

- Yes
- No
- Don’t know/prefer not to answer

Have you ever received information about your risk of developing cancer?

- Yes
- No

If yes, please give details of the information you received and who gave it to you.

Numeracy

As provision of cancer risk includes numbers, we would like to know how you answer the following questions.

Imagine we flip a fair coin 1000 times.

**What is your best guess at how many times the coin would come up heads in 1000 flips?**	

In the UK National Lottery®, the chance of winning a £10 prize is 1%.

**What is your best guess at how many people would win a £10 prize if 1000 people each bought a single ticket to UK NATIONAL LOTTERY®?**	

In the EuroMillions® Lottery, the chance of winning a car is 1 in 1000.

**What percent of the EuroMillions® tickets win a car?**	

Time orientation

For each of the following statements, select the option that best applies to you:

	**Very unlike me**				**Very like me**
**Thinking about the future is pleasant to me.**	Very unlike me				Very like me
**When I want to achieve something I set goals and consider specific means of reaching those goals.**	Very unlike me				Very like me
**Meeting tomorrow's deadlines and doing other necessary work comes before tonight's play.**	Very unlike me				Very like me
**It seems to me that my future plans are pretty well laid out.**	Very unlike me				Very like me
**I think that it seems useless to plan too far ahead because things hardly ever come out the way you planned anyway.**	Very unlike me				Very like me
**If I don't get done on time, I don't worry about it.**	Very unlike me				Very like me
**I try to live one day at a time.**	Very unlike me				Very like me
**I feel that it's more important to enjoy what you are doing than to get the work done on time.**	Very unlike me				Very like me
**I don't do things that are good for me if they don't feel good now.**	Very unlike me				Very like me

Awareness of cancer risk factors

Below are some of the things that can increase a person's chance of developing cancer. How much do you agree that each of these can increase a person's chance of developing cancer?

	Strongly disagree	Disagree	Not sure	Agree	Strongly agree
**Smoking any cigarettes at all**					
**Exposure to another person's cigarette smoke**					
**Drinking more than 1 unit of alcohol a day**					
**Eating less than 5 portions of fruit and vegetables a day**					

**Eating red or processed meat once a day or more**					
**Being overweight (BMI over 25)**					
**Getting sunburnt more than once as a child**					
**Being over 70 years old**					
**Having a close relative with cancer**					
**Infection with HPV (Human Papillomavirus)**					
**Doing less than 30 mins of moderate physical activity 5 times a week**					

Cancer risk perceptions

The five most common preventable cancers for men are lung cancer, colorectal cancer, bladder cancer, kidney cancer and oesophageal cancer. The five most common preventable cancers for women are breast cancer, lung cancer, colorectal cancer, endometrial cancer and kidney cancer.

How likely do you think is it that you will get one of these five cancers at some point in the next 10 years?

Likely Unlikely

On a scale from 0 to 100%, how would you rate the probability that you will develop one of these five cancers in the next 10 years?

How certain are you about your answer to the above question?

Not at all certain Extremely certain

How confident are you that the estimate you have given is accurate, that is, that it reflects your actual risk?

Not at all confident Extremely confident

How do you think your chance of developing one of these five cancers in the next 10 years compares to the average person of your sex and age?

Much lower Much higher

Again, how certain are you about your answer to the above question?

Not at all certain | Extremely certain

How confident are you that the estimate you have given is accurate, that is, that it reflects your actual risk?

Not at all confident | Extremely confident

The way I look after my health means that my odds of getting one of these five cancers in the future are:

Very low | Very high

When I think carefully about my lifestyle, it does seem possible that I could get one of these five cancers.

Strongly disagree | Strongly agree

If I look at myself as if I was a doctor, I realise that my behaviour puts me at risk of getting one of these five cancers.

Strongly disagree | Strongly agree

How worried are you about developing cancer in the future?

Not at all | Extremely

How fearful are you about developing cancer in the future?

Not at all | Extremely

How nervous are you about developing cancer in your lifetime?

Not at all | Extremely

When you think about cancer for a moment, to what extent do you feel fearful?

Not at all | Extremely

When you think about cancer for a moment, to what extent do you feel worried?

Not at all Extremely

When you think about cancer for a moment, to what extent do you feel anxious?

Not at all Extremely

How concerned are you about developing cancer in your lifetime?

Not at all Extremely

How easy is it for you to imagine yourself developing cancer in the future?

Not at all Extremely

I feel very vulnerable to disease.

Strongly disagree Strongly agree

I am confident that I will not get cancer.

Strongly disagree Strongly agree

I would be lying if I said "There is no chance of me getting cancer."

Strongly disagree Strongly agree

My first reaction when I hear of someone getting cancer is "that is could be me someday".

Strongly disagree Strongly agree

For each of the following statements, select the option that best applies to you:

	Not at all	Rarely	Sometimes	Often	A lot
**During the past month, how often have you thought about your own chances of getting cancer?**					
**During the past month, how often have thoughts about your chances of getting cancer affected your mood?**					
**During the past month, how often have thoughts about your chances of getting cancer affected your ability to perform your daily activities?**					

A number of statements which people have used to describe themselves are given below. Please read each statement and then circle the most appropriate number to the right of the statement to indicate how you feel right now, at this moment. There are no right or wrong answers. Do not spend too much time on any one statement but give the answer which seems to describe your present feelings best.

	Not at all	Somewhat	Moderately	Very much
1. I feel calm	1	2	3	4
2. I am tense	1	2	3	4
3. I feel upset	1	2	3	4
4. I am relaxed	1	2	3	4
5. I feel content	1	2	3	4
6. I am worried	1	2	3	4

Maladaptive Behaviours

For each of the following statements, select the option that best applies to you:

	Strongly disagree	Disagree	Neither agree nor disagree	Agree	Strongly agree
**I try not to think about the possibility of developing cancer.** ***[Avoidance]***					
**If you are destined to develop cancer you will, there is really very little you can do about it.** ***[Fatalism]***					
**Given what I know about cancer, I sometimes feel it is almost useless to try to stay healthy.** ***[Hopelessness]***					

As part of your consultation you will also receive information about your cardiovascular disease risk, please now complete these questions.

About you and your health

Have your parents or any brothers or sisters ever had cardiovascular disease?

- Yes
- No
- Don't know/prefer not to answer

Have you ever received information on your risk of developing cardiovascular disease?

- Yes
- No

If yes, please give details of the information you received and who gave it to you.

Cardiovascular risk perceptions

On a scale from 0 to 100%, how would you rate the probability that you will develop cardiovascular disease in the next 10 years?

How certain are you about your answer to the above question?

Not at all certain — Extremely certain

How confident are you that the estimate you have given is accurate, that is, that it reflects your actual risk?

Not at all confident — Extremely confident

About you and your lifestyle

What is your ethnic group? Choose one option that best describes your ethnic group or background.

- White
- Mixed / Multiple ethnic group
- Asian / Asian British
- Black / African / Caribbean / Black British
- Other, please describe

Please complete part of your postcode…

Please enter your highest education level

- No formal education
- Primary education (educated to age 11 or before)
- Secondary education (educated to age 18 or before)
- University education

Are you…

- Female
- Male

How old are you?

How much do you weigh? *[There is the option for Kg or stone]*

What is your height? *[There is the option for meters or feet and inches]*

Do you currently smoke?

- Yes
- No, but I used to
- No, and I never have

How many portions of fruit do you eat on a typical day? *[With images of examples of one portion]*

How many portions of vegetables do you eat in a typical day? *[With images of examples of one portion]*

How many portions of red meat do you eat in a typical week? *[With images of examples of one portion]*

How many portions of processed meat do you eat in a typical week? *[With images of examples of one portion]*

How many hours of physical activity such as brisk walking, cycling, keep fit, aerobics, swimming or jogging, do you do in a typical week?

How many units of alcohol do you drink in a typical day? *[With images of what one unit of alcohol is equivalent to]*

Immediately post-consultation Questionnaire

Now that you have seen some information about the factors that influence risk of cancer and cardiovascular disease, please answer the following questions. As before, there are no right or wrong answers.

About the information you received

Thinking about the risk information and lifestyle advice you received relating to cancer, select the option that best applies to you:

	Strongly disagree	Disagree	Neither agree nor disagree	Agree	Strongly agree
**The risk information was understandable and helped me to decide about decreasing my risk of cancer.**					
**The risk information was trustworthy and helped me to decide about decreasing my risk of cancer.**					
**The risk information was useful and helped me to decide about decreasing my risk of cancer.**					
**The risk information was motivating and helped me to decide about decreasing my risk of cancer.**					
**The risk information was important and helped me to decide about decreasing my risk of cancer.**					
**The risk information was well presented and helped me decide about decreasing my risk of cancer.**					
**The lifestyle advice was understandable and helped me to decide about decreasing my risk of cancer.**					
**The lifestyle advice was trustworthy and helped me to decide about decreasing my risk of cancer.**					
**The lifestyle advice was useful and helped me to decide about decreasing my risk of cancer.**					
**The lifestyle advice was motivating and helped me to decide about decreasing my risk of cancer.**					
**The lifestyle advice was important and helped me to decide about decreasing my risk of cancer.**					
**The lifestyle advice was well presented and helped me to decide about decreasing my risk of cancer.**					

Awareness of cancer risk factors

Below are some of the things that can increase a person's chance of developing cancer. How much do you agree that each of these can increase a person's chance of developing cancer?

	Strongly disagree	Disagree	Not sure	Agree	Strongly agree
**Smoking any cigarettes at all**					
**Exposure to another person's cigarette smoke**					
**Drinking more than 1 unit of alcohol a day**					
**Eating less than 5 portions of fruit and vegetables a day**					
**Eating red or processed meat once a day or more**					
**Being overweight (BMI over 25)**					
**Getting sunburnt more than once as a child**					
**Being over 70 years old**					
**Having a close relative with cancer**					
**Infection with HPV (Human Papillomavirus)**					
**Doing less than 30 mins of moderate physical activity 5 times a week**					

Cancer risk perceptions

The five most common preventable cancers for men are lung cancer, colorectal cancer, bladder cancer, kidney cancer and oesophageal cancer. The five most common preventable cancers for women are breast cancer, lung cancer, colorectal cancer, endometrial cancer and kidney cancer.

How likely do you think is it that you will get one of these five cancers at some point in the next 10 years?

Likely Unlikely

On a scale from 0 to 100%, how would you rate the probability that you will develop one of these five cancers in the next 10 years?

How certain are you about your answer to the above question?

Not at all certain — Extremely certain

How confident are you that the estimate you have given is accurate, that is, that it reflects your actual risk?

Not at all confident — Extremely confident

How do you think your chance of developing one of these five cancers in the next 10 years compares to the average person of your sex and age?

Much lower — Much higher

Again, how certain are you about your answer to the above question?

Not at all certain — Extremely certain

How confident are you that the estimate you have given is accurate, that is, that it reflects your actual risk?

Not at all confident — Extremely confident

The way I look after my health means that my odds of getting one of these five cancers in the future are:

Very low — Very high

When I think carefully about my lifestyle, it does seem possible that I could get one of these five cancers.

Strongly disagree — Strongly agree

If I look at myself as if I was a doctor, I realise that my behaviour puts me at risk of getting one of these five cancers.

Strongly disagree — Strongly agree

How worried are you about developing cancer in the future?

Not at all — Extremely

How fearful are you about developing cancer in the future?

Not at all Extremely

How nervous are you about developing cancer in your lifetime?

Not at all Extremely

When you think about cancer for a moment, to what extent do you feel fearful?

Not at all Extremely

When you think about cancer for a moment, to what extent do you feel worried?

Not at all Extremely

When you think about cancer for a moment, to what extent do you feel anxious?

Not at all Extremely

How concerned are you about developing cancer in your lifetime?

Not at all Extremely

How easy is it for you to imagine yourself developing cancer in the future?

Not at all Extremely

I feel very vulnerable to disease.

Strongly disagree Strongly agree

I am confident that I will not get cancer.

Strongly disagree Strongly agree

I would be lying if I said "There is no chance of me getting cancer."

Strongly disagree Strongly agree

My first reaction when I hear of someone getting cancer is "that could be me someday".

Strongly disagree Strongly agree

A number of statements which people have used to describe themselves are given below. Please read each statement and then circle the most appropriate number to the right of the statement to indicate how you feel right now, at this moment. There are no right or wrong answers. Do not spend too much time on any one statement but give the answer which seems to describe your present feelings best.

	Not at all	Somewhat	Moderately	Very much
1. I feel calm	1	2	3	4
2. I am tense	1	2	3	4
3. I feel upset	1	2	3	4
4. I am relaxed	1	2	3	4
5. I feel content	1	2	3	4
6. I am worried	1	2	3	4

Behavioural Intentions

We are interested in your views about making changes to your lifestyle in the future. Please read each of the statements below and select the most appropriate box to indicate your view.

	Strongly disagree	Disagree	Somewhat disagree	Neither agree or disagree	Somewhat agree	Agree	Strongly agree
**I am determined to do everything I can to avoid getting cancer in the future.**							
**I am committed to engaging in behaviours that protect me against getting cancer in the future.**							
**I fully intend to have a lifestyle that will prevent me from getting cancer in the future.**							
**I will try to do all I can to avoid getting cancer in the future.**							

	Strongly disagree	Disagree	Neither agree or disagree	Agree	Strongly agree	Not applicable
**I intend to be more physically active in the next 3 months**						
**I intend to eat a more fruit and vegetables in the next 3 months**						
**I intend to drink less alcohol in the next 3 months**						
**I intend to eat less red meat in the next 3 months**						
**I intend to eat less processed meat in the next 3 months**						
**I intend to cut down or quit in the next 3 months smoking**						
**I intend to try and lose weight in the next 3 months**						

Maladaptive behaviours

For each of the following statements, select the option that best applies to you:

	Strongly disagree	Disagree	Neither agree nor disagree	Agree	Strongly agree
**I try not to think about the possibility of developing cancer. *[Avoidance]***					
**If you are destined to develop cancer you will, there is really very little you can do about it. *[Fatalism]***					
**Given what I know about cancer, I sometimes feel it is almost useless to try to stay healthy. *[Hopelessness]***					

Response efficacy

Please read each of the statements below and tick the most appropriate box to indicate how much you agree or disagree with each statement.

If I were to be active at a moderate intensity for at least 30 minutes per day on at least 5 days a week, I would reduce my risk of developing cancer.

- Strongly disagree
- Disagree
- Neither agree nor disagree
- Agree
- Strongly agree

Being physically active is effective in preventing cancer.

- Strongly disagree
- Disagree
- Neither agree nor disagree
- Agree
- Strongly agree

If I am physically active, I am less likely to develop cancer.

- Strongly disagree
- Disagree
- Neither agree nor disagree
- Agree
- Strongly agree

If I were to consume 5 servings of fruit and vegetables each day, I would reduce my risk of developing cancer.

- Strongly disagree
- Disagree
- Neither agree nor disagree
- Agree
- Strongly agree

Eating a healthy diet is effective in preventing cancer.

- Strongly disagree
- Disagree
- Neither agree nor disagree
- Agree
- Strongly agree

If I eat a healthy diet, I am less likely to develop cancer.

- Strongly disagree
- Disagree
- Neither agree nor disagree
- Agree
- Strongly agree

Self-efficacy

I would like to be physically active but I don't know if I can.

- Strongly disagree
- Disagree
- Neither agree nor disagree
- Agree
- Strongly agree

I feel confident in my ability to be active at a moderate intensity for at least 30 minutes per day on at least 5 days a week.

- Strongly disagree
- Disagree
- Neither agree nor disagree
- Agree
- Strongly agree

I am confident that I could be physically active if I wanted to.

- Strongly disagree
- Disagree
- Neither agree nor disagree
- Agree
- Strongly agree

I would like to eat more fruit and vegetables but I don't know if I can.

- Strongly disagree
- Disagree
- Neither agree nor disagree
- Agree
- Strongly agree

I feel confident in my ability to consume 5 servings of fruit and vegetables each day.

- Strongly disagree
- Disagree
- Neither agree nor disagree
- Agree
- Strongly agree

I am confident that I could eat more fruit and vegetables if I wanted to.

- Strongly disagree
- Disagree
- Neither agree nor disagree
- Agree
- Strongly agree

As part of your consultation you were also given your risk of cardiovascular disease (heart attack or stroke). Please now complete these questions.

Cardiovascular disease risk perceptions

On a scale from 0 to 100%, how would you rate the probability that you will develop cardiovascular disease in the next 10 years?

How certain are you about your answer to the above question?

Not at all certain Extremely certain

How confident are you that the estimate you have given is accurate, that is, that it reflects your actual risk?

Not at all confident Extremely confident

Cardiovascular disease behavioural intentions

We are interested in your views about making changes to your lifestyle in the future. Please read each of the statements below and select the most appropriate box to indicate your view.

	Strongly disagree	Disagree	Somewhat disagree	Neither agree or disagree	Somewhat agree	Agree	Strongly agree
**I am determined to do everything I can to avoid getting cardiovascular disease in the future.**							
**I am committed to engaging in behaviours that protect me against getting cardiovascular disease in the future.**							
**I fully intend to have a lifestyle that will prevent me from getting cardiovascular disease in the future.**							
**I will try to do all I can to avoid getting cardiovascular disease in the future.**							

	Strongly disagree	Disagree	Neither agree or disagree	Agree	Strongly agree	Not applicable
**I intend to be more physically active in the next 3 months**						
**I intend to eat a more fruit and vegetables in the next 3 months**						
**I intend to drink less alcohol in the next 3 months**						
**I intend to eat less red meat in the next 3 months**						
**I intend to eat less processed meat in the next 3 months**						
**I intend to cut down or quit in the next 3 months smoking**						
**I intend to try and lose weight in the next 3 months**						

Medication

Did the healthcare professional discuss you starting taking statins (cholesterol lowering medication) at your consultation?

Yes ☐

No ☐

I don't know ☐

Are you planning to start taking this medication?

Yes ☐

No ☐

Not sure ☐

Please explain in a few words why you made that decision…

Did the healthcare professional discuss you starting taking blood pressure lowering medication at your consultation?

Yes ☐

No ☐

I don't know ☐

Are you planning to start taking this medication?

Yes ☐

No ☐

Not sure ☐

Please explain in a few words why you made that decision…

3-month follow-up Questionnaire

Thank you again for taking part in this study. Please answer every question. If you are uncertain about how to answer a question, then please select the closest option. There are no right or wrong answers.

About you and your lifestyle

How much do you weigh? *[There is the option for Kg or stone]*

Do you currently smoke?

- Yes
- No, but I used to
- No, and I never have

How many portions of fruit do you eat on a typical day? *[With images of examples of one portion]*

How many portions of vegetables do you eat in a typical day? *[With images of examples of one portion]*

How many portions of red meat do you eat in a typical week? *[With images of examples of one portion]*

How many portions of processed meat do you eat in a typical week? *[With images of examples of one portion]*

How many hours of physical activity such as brisk walking, cycling, keep fit, aerobics, swimming or jogging, do you do in a typical week?

How many units of alcohol do you drink in a typical day? *[With images of what one unit of alcohol is equivalent to]*

Awareness of cancer risk factors

Below are some of the things that can increase a person's chance of developing cancer. How much do you agree that each of these can increase a person's chance of developing cancer?

	Strongly disagree	Disagree	Not sure	Agree	Strongly agree
**Smoking any cigarettes at all**					
**Exposure to another person's cigarette smoke**					
**Drinking more than 1 unit of alcohol a day**					
**Eating less than 5 portions of fruit and vegetables a day**					
**Eating red or processed meat once a day or more**					
**Being overweight (BMI over 25)**					
**Getting sunburnt more than once as a child**					
**Being over 70 years old**					
**Having a close relative with cancer**					
**Infection with HPC (Human Papillomavirus)**					
**Doing less than 30 mins of moderate physical activity 5 times a week**					

Cancer risk perceptions

The five most common preventable cancers for men are lung cancer, colorectal cancer, bladder cancer, kidney cancer and oesophageal cancer. The five most common preventable cancers for women are breast cancer, lung cancer, colorectal cancer, endometrial cancer and kidney cancer.

How likely do you think is it that you will get one of these five cancers at some point in the next 10 years?

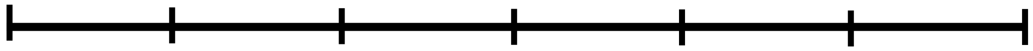

Likely Unlikely

On a scale from 0 to 100%, how would you rate the probability that you will develop one of these five cancers in the next 10 years?

How certain are you about your answer to the above question?

Not at all certain Extremely certain

How confident are you that the estimate you have given is accurate, that is, that it reflects your actual risk?

Not at all confident Extremely confident

How do you think your chance of developing one of these five cancers in the next 10 years compares to the average person of your sex and age?

Much lower Much higher

Again, how certain are you about your answer to the above question?

Not at all certain Extremely certain

How confident are you that the estimate you have given is accurate, that is, that it reflects your actual risk?

Not at all confident Extremely confident

The way I look after my health means that my odds of getting one of these five cancers in the future are:

Very low Very high

When I think carefully about my lifestyle, it does seem possible that I could get one of these five cancers.

Strongly disagree Strongly agree

If I look at myself as if I was a doctor, I realise that my behaviour puts me at risk of getting one of these five cancers.

Strongly disagree Strongly agree

How worried are you about developing cancer in the future?

Not at all Extremely

How fearful are you about developing cancer in the future?

Not at all Extremely

How nervous are you about developing cancer in your lifetime?

Not at all Extremely

When you think about cancer for a moment, to what extent do you feel fearful?

Not at all Extremely

When you think about cancer for a moment, to what extent do you feel worried?

Not at all Extremely

When you think about cancer for a moment, to what extent do you feel anxious?

Not at all Extremely

How concerned are you about developing cancer in your lifetime?

Not at all Extremely

How easy is it for you to imagine yourself developing cancer in the future?

Not at all Extremely

I feel very vulnerable to disease.

Strongly disagree Strongly agree

I am confident that I will not get cancer.

Strongly disagree Strongly agree

I would be lying if I said "There is no chance of me getting cancer."

Strongly disagree Strongly agree

My first reaction when I hear of someone getting cancer is “that could be me someday”.

Strongly disagree Strongly agree

For each of the following statements, select the option that best applies to you:

	Not at all	Rarely	Sometimes	Often	A lot
**During the past month, how often have you thought about your own chances of getting cancer?**					
**During the past month, how often have thoughts about your chances of getting cancer affected your mood?**					
**During the past month, how often have thoughts about your chances of getting cancer affected your ability to perform your daily activities?**					

A number of statements which people have used to describe themselves are given below. Please read each statement and then circle the most appropriate number to the right of the statement to indicate how you feel right now, at this moment. There are no right or wrong answers. Do not spend too much time on any one statement but give the answer which seems to describe your present feelings best.

	Not at all	Somewhat	Moderately	Very much
1. I feel calm	1	2	3	4
2. I am tense	1	2	3	4
3. I feel upset	1	2	3	4
4. I am relaxed	1	2	3	4
5. I feel content	1	2	3	4
6. I am worried	1	2	3	4

Maladaptive behaviours

For each of the following statements, select the option that best applies to you:

	Strongly disagree	Disagree	Neither agree nor disagree	Agree	Strongly agree
**I try not to think about the possibility of developing cancer. *[Avoidance]***					
**If you are destined to develop cancer you will, there is really very little you can do about it. *[Fatalism]***					
**Given what I know about cancer, I sometimes feel it is almost useless to try to stay healthy. *[Hopelessness]***					

As part of your recent consultation you were also given your risk of cardiovascular disease (heart attack or stroke). Please now complete these questions.

Cardiovascular disease risk perceptions

On a scale from 0 to 100%, how would you rate the probability that you will develop cardiovascular disease in the next 10 years?

How certain are you about your answer to the above question?

Not at all certain — Extremely certain

How confident are you that the estimate you have given is accurate, that is, that it reflects your actual risk?

Not at all confident — Extremely confident

Medication

Have you had a discussion with a healthcare professional about starting statins (cholesterol lowering medication), either in your NHS Health Check or since then?

Yes ☐

No ☐

If yes,

Did you start taking them?

Yes ☐

No ☐

Please explain in a few words why you made that decision….

If yes,

Are you still taking them?

Yes ☐

No ☐

If no, please explain briefly why you stopped taking them….

Have you had a discussion with a healthcare professional about starting blood pressure medication, either in your NHS Health Check or since then?

Yes ☐

No ☐

If yes,

Did you start taking them?

Yes ☐

No ☐

Please explain in a few words why you made that decision….

Are you still taking them?

Yes ☐

No ☐

If no, please explain briefly why you stopped taking them….
