# Supplementary material for: Incorporating a brief intervention for personalised cancer risk assessment to promote behaviour change into primary care: a multi-methods pilot study
Source: BMC Public Health. 2021 Jan 23;21:205. doi: 10.1186/s12889-021-10210-3 (PMC7824918; doi:10.1186/s12889-021-10210-3)
Supplement: Supplementary file 7 — Additional File 7. Questionnaires for standard NHS Health Check or chronic disease review group. [file 12889_2021_10210_MOESM7_ESM.pdf]

I-CaPP Programme- Pilot Study

Exploring communication of disease risk in primary care

Questionnaires for standard NHS Health Check or chronic disease review group

[Note that this content was delivered online so the formatting looked different to in this document and participants did not see the subheadings highlighted in blue. The participants also had clear instructions on how to respond to each question depending on the format e.g. radio buttons/drop-down lists]

Baseline Questionnaire

Thank you for agreeing to complete this questionnaire. Please answer every question. If you are uncertain about how to answer a question, then please select the closest option. There are no right or wrong answers.

About you and your health

How would you rate your general health?

- Very good
- Quite good
- Neither good nor poor
- Quite poor
- Poor

Have your parents or any brothers or sisters ever had cardiovascular disease?

- Yes
- No
- Don't know/prefer not to answer

Have you ever received information about your risk of developing cardiovascular disease?

- Yes
- No

If yes, please give details of the information you received and who gave it to you.

Numeracy

As provision of cardiovascular risk includes numbers, we would like to know how you answer the following questions.

Imagine we flip a fair coin 1000 times.

**What is your best guess at how many times the coin would come up heads in 1000 flips?**	

In the UK National Lottery®, the chance of winning a £10 prize is 1%.

**What is your best guess at how many people would win a £10 prize if 1000 people each bought a single ticket to UK NATIONAL LOTTERY®?**	

In the EuroMillions® Lottery, the chance of winning a car is 1 in 1000.

**What percent of the EuroMillions® tickets win a car?**	

Time orientation

For each of the following statements, select the option that best applies to you:

	**Very unlike me**				**Very like me**
**Thinking about the future is pleasant to me.**	Very unlike me				Very like me
**When I want to achieve something I set goals and consider specific means of reaching those goals.**	Very unlike me				Very like me
**Meeting tomorrow's deadlines and doing other necessary work comes before tonight's play.**	Very unlike me				Very like me
**It seems to me that my future plans are pretty well laid out.**	Very unlike me				Very like me
**I think that it seems useless to plan too far ahead because things hardly ever come out the way you planned anyway.**	Very unlike me				Very like me
**If I don't get done on time, I don't worry about it.**	Very unlike me				Very like me
**I try to live one day at a time.**	Very unlike me				Very like me
**I feel that it's more important to enjoy what you are doing than to get the work done on time.**	Very unlike me				Very like me
**I don't do things that are good for me if they don't feel good now.**	Very unlike me				Very like me

Cardiovascular risk perceptions

On a scale from 0 to 100%, how would you rate the probability that you will develop cardiovascular disease in the next 10 years?

How certain are you about your answer to the above question?

Not at all certain — Extremely certain

How confident are you that the estimate you have given is accurate, that is, that it reflects your actual risk?

Not at all confident — Extremely confident

About you and your lifestyle

What is your ethnic group? Choose one option that best describes your ethnic group or background.

- White
- Mixed / Multiple ethnic group
- Asian / Asian British
- Black / African / Caribbean / Black British
- Other, please describe

Please complete part of your postcode…

Please enter your highest education level

- No formal education
- Primary education (educated to age 11 or before)
- Secondary education (educated to age 18 or before)
- University education

Are you…

- Female
- Male

How old are you?

How much do you weigh? *[There is the option for Kg or stone]*

What is your height? *[There is the option for meters or feet and inches]*

Do you currently smoke?

- Yes
- No, but I used to
- No, and I never have

How many portions of fruit do you eat on a typical day? *[With images of examples of one portion]*

How many portions of vegetables do you eat in a typical day? *[With images of examples of one portion]*

How many portions of red meat do you eat in a typical week? *[With images of examples of one portion]*

How many portions of processed meat do you eat in a typical week? *[With images of examples of one portion]*

How many hours of physical activity such as brisk walking, cycling, keep fit, aerobics, swimming or jogging, do you do in a typical week?

How many units of alcohol do you drink in a typical day? *[With images of what one unit of alcohol is equivalent to]*

Immediately post-consultation Questionnaire

Now that you have been given some information about your risk of cardiovascular disease (heart attack or stroke) and ways to reduce that, please answer the following questions. As before, there are no right or wrong answers.

Cardiovascular disease risk perceptions

On a scale from 0 to 100%, how would you rate the probability that you will develop cardiovascular disease in the next 10 years?

How certain are you about your answer to the above question?

Not at all certain — Extremely certain

How confident are you that the estimate you have given is accurate, that is, that it reflects your actual risk?

Not at all confident — Extremely confident

Behavioural Intentions

We are interested in your views about making changes to your lifestyle in the future. Please read each of the statements below and select the most appropriate box to indicate your view.

	Strongly disagree	Disagree	Somewhat disagree	Neither agree or disagree	Somewhat agree	Agree	Strongly agree
**I am determined to do everything I can to avoid getting cardiovascular disease in the future.**							
**I am committed to engaging in behaviours that protect me against getting cardiovascular disease in the future.**							
**I fully intend to have a lifestyle that will prevent me from getting cardiovascular disease in the future.**							
**I will try to do all I can to avoid getting cardiovascular disease in the future.**							

	Strongly disagree	Disagree	Neither agree or disagree	Agree	Strongly agree	Not applicable
**I intend to be more physically active in the next 3 months**						
**I intend to eat a more fruit and vegetables in the next 3 months**						
**I intend to drink less alcohol in the next 3 months**						
**I intend to eat less red meat in the next 3 months**						
**I intend to eat less processed meat in the next 3 months**						
**I intend to cut down or quit in the next 3 months smoking**						
**I intend to try and lose weight in the next 3 months**						

Medication

Did the healthcare professional discuss you starting taking statins (cholesterol lowering medication) at your consultation?

Yes ☐

No ☐

I don’t know ☐

Are you planning to start taking this medication?

Yes ☐

No ☐

Not sure ☐

Please explain in a few words why you made that decision…

Did the healthcare professional discuss you starting taking blood pressure lowering medication at your consultation?

Yes ☐

No ☐

I don’t know ☐

Are you planning to start taking this medication?

Yes ☐

No ☐

Not sure ☐

Please explain in a few words why you made that decision…

3-month follow-up Questionnaire

Now that you have been given some information about your risk of cardiovascular disease and ways to reduce that, please answer the following questions. As before, there are no right or wrong answers.

Cardiovascular disease risk perceptions

On a scale from 0 to 100%, how would you rate the probability that you will develop cardiovascular disease in the next 10 years?

How certain are you about your answer to the above question?

Not at all certain | Extremely certain

How confident are you that the estimate you have given is accurate, that is, that it reflects your actual risk?

Not at all confident | Extremely confident

Medication

Have you had a discussion with a healthcare professional about starting statins (cholesterol lowering medication), either in your NHS Health Check or since then?

Yes ☐

No ☐

If yes,

Did you start taking them?

Yes ☐

No ☐

Please explain in a few words why you made that decision….

If yes,

Are you still taking them?

Yes ☐

No ☐

If no, please explain briefly why you stopped taking them….

Have you had a discussion with a healthcare professional about starting blood pressure medication, either in your NHS Health Check or since then?

Yes ☐

No ☐

If yes,

Did you start taking them?

Yes ☐

No ☐

Please explain in a few words why you made that decision….

Are you still taking them?

Yes ☐

No ☐

If no, please explain briefly why you stopped taking them….

About you and your lifestyle

How much do you weigh? *[There is the option for Kg or stone]*

Do you currently smoke?

- Yes
- No, but I used to
- No, and I never have

How many portions of fruit do you eat on a typical day? *[With images of examples of one portion]*

How many portions of vegetables do you eat in a typical day? *[With images of examples of one portion]*

How many portions of red meat do you eat in a typical week? *[With images of examples of one portion]*

How many portions of processed meat do you eat in a typical week? *[With images of examples of one portion]*

How many hours of physical activity such as brisk walking, cycling, keep fit, aerobics, swimming or jogging, do you do in a typical week?

How many units of alcohol do you drink in a typical day? *[With images of what one unit of alcohol is equivalent to]*

