# Supplementary material for: Incorporating a brief intervention for personalised cancer risk assessment to promote behaviour change into primary care: a multi-methods pilot study
Source: BMC Public Health. 2021 Jan 23;21:205. doi: 10.1186/s12889-021-10210-3 (PMC7824918; doi:10.1186/s12889-021-10210-3)
Supplement: Supplementary file 8 — Additional File 8. Psychological outcomes and risk factor awareness within the groups receiving the intervention. [file 12889_2021_10210_MOESM8_ESM.pdf]

Additional File 8. Psychological outcomes and risk factor awareness within the groups receiving the intervention

		Baseline<br>(n=50)		Immediate follow-up<br>(n=35)		3 month<br>(n=30)
	N	**Mean (SD)**	**n**	**Change from baseline Mean (SD)**	**n**	**Change from baseline Mean (SD)**
**Risk Perception**						
Perceived AR	45	29.3 (22.8)	31	-4.1 (-14.0 to 5.8)	22	-0.52 (-8.45 to 7.41)
Conviction of Perceived AR	48	3.58 (1.7)	32	0.33 (-0.45 to 1.1)	26	0.33 (-0.31 to 0.96)
Perceived RR	50	3.48 (1.2)	35	0.2 (-0.24 to 0.64)	29	0.03 (-0.51 to 0.58)
Conviction of Perceived RR	47	3.86 (1.6)	33	0.20 (-0.41 to 0.82)	24	0.38 (-0.25 to 1.00)
**Risk Perception**						
Perceived AR	45	29.3 (22.8)	31	-4.1 (-14.0 to 5.8)	22	-0.52 (-8.45 to 7.41)
Conviction of Perceived AR	48	3.58 (1.7)	32	0.33 (-0.45 to 1.1)	26	0.33 (-0.31 to 0.96)
Perceived RR	50	3.48 (1.2)	35	0.2 (-0.24 to 0.64)	29	0.03 (-0.51 to 0.58)
Conviction of Perceived RR	47	3.86 (1.6)	33	0.20 (-0.41 to 0.82)	24	0.38 (-0.25 to 1.00)
**Maladaptive Coping Behaviour**	48	9.3 (2.0)	35	0.35 (-0.47 to 1.18)	29	0.45 (-0.39 to 1.28)
**Cancer Worry**	46	4.8 (2.0)	---	---	25	0.12 (-0.40 to 0.64)
**Anxiety**	45	12.1 (2.2)	34	0.75 (-0.14 to 1.64)	25	0.64 (-0.22 to 1.50)
		**% (95% CI)**		**% (95% CI)**		**% (95% CI)**
**Absolute risk accuracy**	45		31		24	
Accurate (±5%)		17.8 (8.9 to 32.4)		25.8 (12.9 to 44.9)		25.0 (11.0 to 47.4)
Underestimate		4.4 (1.1 to 16.9)		0 (0)		0 (0)
Overestimate		77.8 (62.8 to 87.9)		74.2 (55.1 to 87.1)		75.0 (52.6 to 89.0)
**Comparative risk accuracy**	50		35		29	
Accurate		36.0 (23.6 to 50.6)		25.7 (13.5 to 43.4)		41.4 (24.3 to 60.8)
Underestimate		48.0 (34.2 to 62.1)		51.4 (34.5 to 68.0)		44.9 (27.2 to 63.9)
Overestimate		16.0 (8.0 to 29.4)		22.9 (11.4 to 40.5)		13.8 (4.9 to 33.0)
